# Coexistence of Lateral and Co-Tuned Inhibitory Configurations in Cortical Networks

**DOI:** 10.1371/journal.pcbi.1002161

**Published:** 2011-10-06

**Authors:** Robert B. Levy, Alex D. Reyes

**Affiliations:** Center for Neural Science, New York University, New York, New York, United States of America; Indiana University, United States of America

## Abstract

The responses of neurons in sensory cortex depend on the summation of excitatory and inhibitory synaptic inputs. How the excitatory and inhibitory inputs scale with stimulus depends on the network architecture, which ranges from the lateral inhibitory configuration where excitatory inputs are more narrowly tuned than inhibitory inputs, to the co-tuned configuration where both are tuned equally. The underlying circuitry that gives rise to lateral inhibition and co-tuning is yet unclear. Using large-scale network simulations with experimentally determined connectivity patterns and simulations with rate models, we show that the spatial extent of the input determined the configuration: there was a smooth transition from lateral inhibition with narrow input to co-tuning with broad input. The transition from lateral inhibition to co-tuning was accompanied by shifts in overall gain (reduced), output firing pattern (from tonic to phasic) and rate-level functions (from non-monotonic to monotonically increasing). The results suggest that a single cortical network architecture could account for the extended range of experimentally observed response types between the extremes of lateral inhibitory versus co-tuned configurations.

## Author Summary

The cerebral cortex contains a network of electrically active cells (neurons) interconnected by synapses, which may be excitatory (tending to increase activity) or inhibitory. Network activity, i.e., the ensemble of activity patterns of the individual cells, is driven by input from the sense organs, and creates an internal representation of features of the outside world. In auditory cortex, sound frequency (pitch) is encoded by the physical location of activity in the network. Thus, connections among cells at various distances may blur or sharpen the frequency representation. Recent work in living animals has yielded conflicting results: sharpening of responses via lateral inhibition in some cases, versus balanced excitation and inhibition (co-tuning) in others. It was previously unknown whether a single cortical network architecture could account for this spectrum of findings. Here, computer simulations based on experimental data reveal that this is indeed the case. Varying input to the network causes smooth transitions between lateral inhibition and co-tuning, accompanied by changes in the strength and timing of the responses. Diverse input-dependent response patterns in a single network may be a general mechanism enabling the brain to process a wide range of sensory information under various conditions.

## Introduction

The firing properties and receptive fields of neurons in sensory cortex are heterogeneous and can vary both quantitatively and qualitatively with changing stimuli. The diverse responses are well exemplified in the primary auditory cortex (A1), where firing ranges from phasic (only at stimulus onset) to tonic (for the duration of a stimulus [1], [2], changes monotonically or non-monotonically with intensity [3], [4], and is often evoked selectively with complex stimuli [5]. Many receptive field properties are not simply inherited from presynaptic input from the thalamus but are shaped by interaction of local excitatory and inhibitory neurons in cortical circuits [6], [7], [8]. The processes governing these interactions are under some debate but are postulated to depend on the network architecture, which may range from the lateral inhibitory network configuration where excitatory inputs are more narrowly tuned than inhibitory inputs, to the co-tuned configuration where both are tuned equally. A1, because of its tonotopic organization, is an ideal system for examining how sensory stimuli are represented in the temporal and spatial interaction of principal cells and interneurons [9], [10]; c.f. [11].

Intracellular recordings *in vivo* have begun to explore mechanisms underlying the diversity of neuronal receptive field properties (for review, see [12]). Though some studies indicate that evoked excitatory and inhibitory conductances are co-tuned [13], [14], others using very similar conditions have found that co-tuning is only approximate or that there is significant lateral inhibition [15], [16], and that the balance may shift during postnatal development [16], [17]. Moreover, many of the response properties such as two-tone suppression and intensity tuning are more consistent with some degree of lateral inhibition [4], [5], [8], [12], [18], [19]. There is a similar debate in the visual system as to the extent to which lateral inhibition in cortex underlies extrareceptive field properties [20], [21].

The cortical circuitry and synaptic properties that underlie co-tuning and lateral inhibition are poorly understood. Lateral inhibition could result from greater sensitivity of inhibitory cells to input, greater convergence of presynaptic (e.g., thalamic) input onto inhibitory versus excitatory cells, or broader spread of intracortical inhibition versus excitation. Canonical circuits typically consist of excitatory pyramidal (P) cells and fast-spiking (FS) interneurons. In layers 2/3 and 4, both P and FS cells receive direct thalamic inputs [22], [23], [24]. FS cells are distinguished from other interneurons by non-adapting high frequency firing [25], morphology [26], [27], [28], and synaptic dynamics [29], [30], suggesting a distinct functional role in sensory information processing.

Here, we performed simulations in a large network model and with rate models to determine how the excitatory and inhibitory drive to P cells changes with stimulus. The patterns of connections and synaptic properties between excitatory P and FS inhibitory cells were based on experimental data. The simulations indicated that the same network generated both lateral inhibition and co-tuning: shifting between them is accomplished simply by changing the spatial distribution of the thalamic input. Therefore, a single, hardwired network potentially is consistent with many of the diverse response patterns previously reported *in vivo*.

## Results

### Model parameters

The broad goal was to build a detailed model of the pattern of stimulus-driven cortical activity. For simplicity, the parameters of the model were taken from published [31], [32], [33] and unpublished (Levy and Reyes, in preparation) experimental data. We stress, however, that the main result - that lateral inhibition and co-tuning coexist in the same network - is robust and does not depend on details of the network connectivity, provided that the inhibition is strong enough (as shown below).

**Figure 1:**
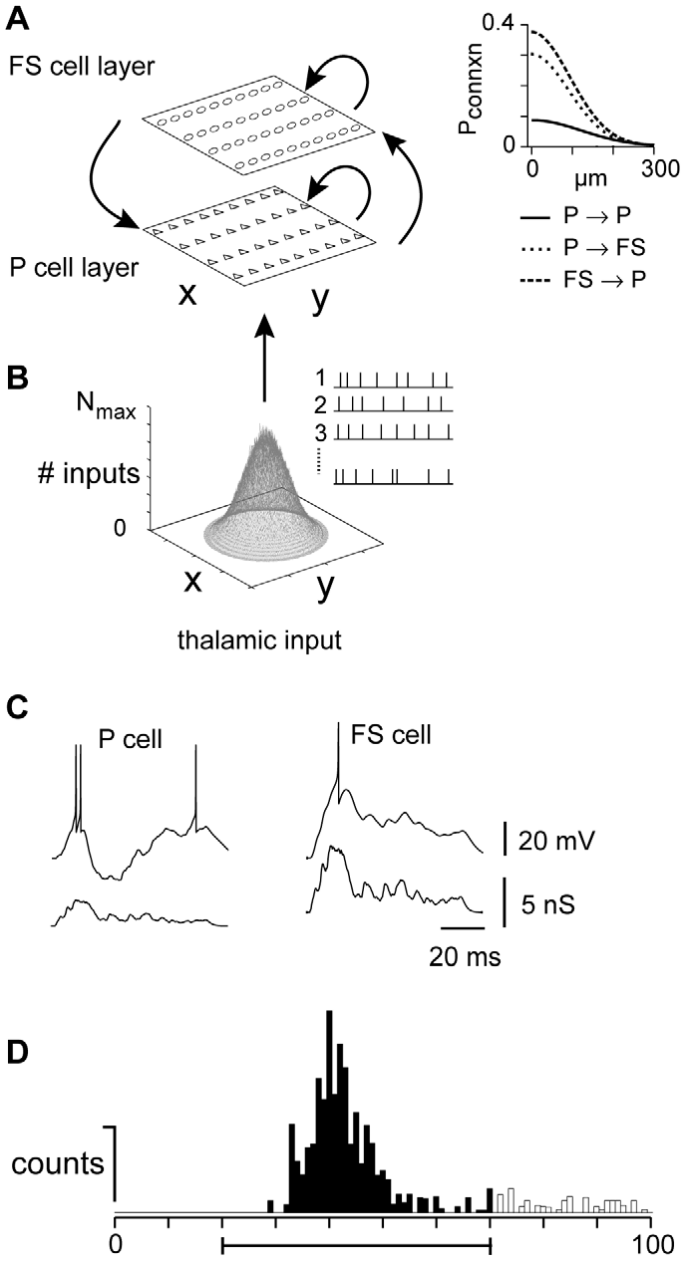
Model schematics. **A**, Left, Network is a sheet of neurons with a pyramidal (P) and a fast spiking (FS) cell layer. Arrows depict connections between and across layers. Right, Gaussian connectivity profiles were fit to experimental data (not shown) for connection probability (abscissa) versus intersomatic distance (ordinate). See Results for parameter values. **B**, The average number of inputs that any P or FS cell received from the thalalmus was Gaussian distributed, with peak value of N_max_. Inset, example thalamic cell trains. **C**, Thalamic conductance input (bottom) and associated voltage response (top) of representative P (left) and FS (right) cells. **D**, Post-stimulus time histogram compiled from spike trains of P cells in the center of the network. Firing was quantified as the number of counts within a 50 ms time interval from the stimulus onset divided by the number of trials (bar, filled portion of the histogram).

Neurons in sensory cortex are connected to their neighbors with a relatively low probability. The connection rates decrease with distance between cells, as the local axonal and dendritic arborizations are confined within several hundred µm of the soma. In cortical layers 2/3 and 4, the thalamorecipient layers of auditory cortex, our experimental data on connection probability versus distance between somata for each connection type (P→P, P→FS, FS→P) were fitted with a Gaussian function (Fig. 1A, right; Methods, equation 1), which was chosen for computational efficiency and to put our findings in the context of the theoretical literature (c.f. [34]). The radial spread of connectivity (σ), i.e., the euclidean distance between cell bodies in the plane of the slice, was 145 µm (P→P), 92 µm (P→FS, FS→P) with peaks of 0.10, 0.30, 0.39, respectively (Fig. 1A, right). Using these connection profiles, we constructed a network sheet of 160×160 P and 32×32 FS cells.

The synaptic and intrinsic membrane properties were also based on experimental data [31], [32], [33], [35]. Measured values for synapse strength (peak amplitude of excitatory and inhibitory postsynaptic potentials (EPSPs/IPSPs) and short-term plasticity were used as parameter values for the phenomenological model of short-term synaptic plasticity in the simulations (Table S2; [36], [37]). The synaptic pathways examined here exhibit strong short-term depression (governed by *U* and *τ_rec_*, Supporting Text S1 and Table S2). Unitary synapse strength, unlike connection probability, was not correlated with distance between cells (c.f. [32], [38]). Therefore the unitary synaptic amplitudes (*A*, Table S2) for each cell type applied at all distances.

The intrinsic membrane properties of P and FS cells used in the simulations are listed in Table S1. We used the adaptive exponential integrate and fire model with variables adjusted appropriately to reproduce the P and FS cell firing behaviors (Fig. 1C; [39]; see Methods).

**Figure 2:**
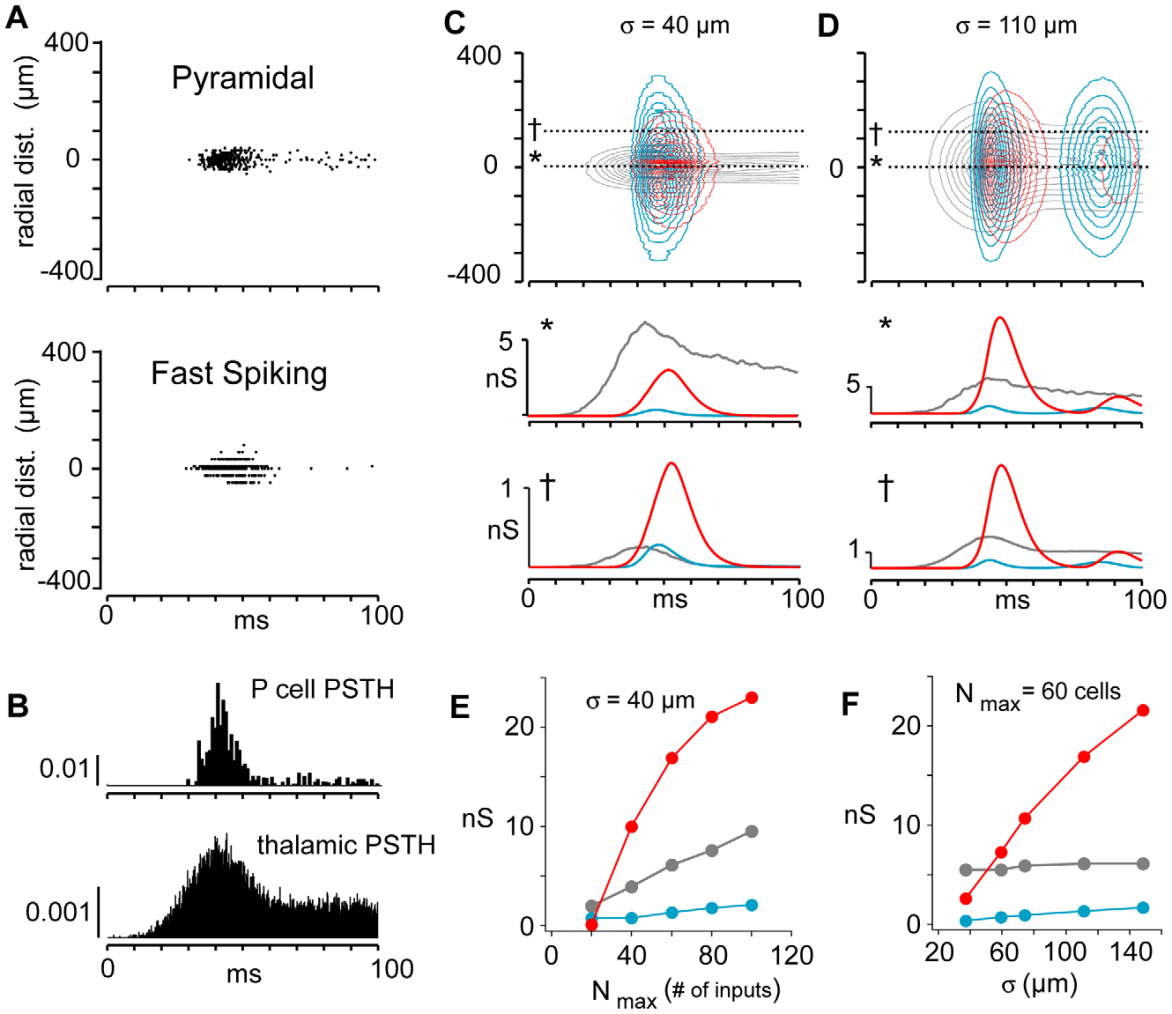
Spatiotemporal profiles of firing and synaptic conductances. **A**, Spike rasters obtained with simulations on a 2 dimensional network of P (top) and FS (bottom) cells. Rasters are arranged according to neurons' symmetric radial distance from the center of the network. Each line is from a representative neuron at a given radial distance and is compiled from 50 sweeps. **B**, Post-stimulus time histograms of P cell (top) and thalamic cell (bottom) populations. **C**, top, Normalized contour plots showing spatiotemporal profiles of thalamic (gray), recurrent excitatory (cyan), and FS inhibitory synaptic conductances (red) evoked in P cells. Temporal profiles of conductances evoked in a P cell at (*, middle) and away (†, bottom) from the center. Thalamic input was narrow (σ = 40 µm). **D**, same as in **C** but for a wider thalamic input (σ = 110 µm). **E**, left, plot of peak conductances (color code as above) vs the number of thalamic inputs N_max_ for σ = 40 µm. **F**, peak conductance vs σ for N_max_  = 60.

The external input to the network was from the auditory thalamus, i.e., the ventral division of the medial geniculate body (MGv). For simplicity, each input was modeled as a sequence of spikes arriving at a specified frequency (train duration  = 100 ms; Fig. 1B). The firing of the thalamic neurons was phasic-tonic (Fig. 2B, bottom; see Methods) as was observed in intact animals [6] though other patterns produced qualitatively similar results (data not shown). The firing rate of thalamic neurons and the number of thalamic cells synapsing onto each cortical cell in the network were Gaussian distributed in space (parameterized by the maximum number of inputs N_max_ each neuron can receive and by the standard deviation σ (in µm; see Methods). Cells in the center of the network received the most inputs (N_max_ = 50**–**150); i.e., the thalamic afferents were maximally active here because the stimulus was assumed to represent the preferred frequency for the center cell. The amplitudes, time course, and short-term dynamics of thalamic inputs were adjusted separately for the P and FS populations (Fig. 1C; Table S2), based on experimental data from auditory cortex [8], [40]; Schiff and Reyes, unpublished data). The thalamic inputs to both P and FS cells exhibited short-term depression (Table S2).

### Shifts in excitatory and inhibitory balance with changing stimuli: Simulations

Several salient features emerge from the simulations. In general, the P and FS cells tended to fire most at the onset of the stimulus (Fig. 2A, top, middle; rasters are compiled from 50 sweeps and arranged according to neurons' radial distance from the center of the 2D network). As observed in awake animals, the P cell firing was more transient than that of thalamic inputs (Fig. 2B; [6]). The transient nature of the P and FS cell firing was due to depressing thalamic inputs (Fig. 1C). The firing pattern of P cells was further shaped by the spatiotemporally complex combination of excitatory input from the thalamus, inhibitory input from FS cells, and excitatory input from neighboring P cells. The normalized contour plot of the synaptic conductances (Fig. 2C, top) shows that the spatial distribution of input to the P cells is variable: the thalamic input (gray) was restricted to the neurons near the center, while the inhibitory inputs from FS cells (red) and recurrent excitatory inputs (cyan) appeared later and were broader.

The timing and relative magnitude of the synaptic components can be seen by focusing on the inputs to the P neuron at the center of the network (* in Fig. 2C, top & middle). A slice through the center of the contour plot shows that the (non-normalized) thalamic input arrives first, causing the initial firing in both the P (Fig. 2A, top) and FS (Fig. 2A, middle) cell populations. After a short delay, the inhibitory input (red, Fig. 2C, middle) from the FS cells appeared followed by the excitatory input from other P cells (cyan). Inhibition was transient due to a combination of transiently firing FS cells (Fig. 2A, bottom) and depressing IPSPs [33]. The recurrent excitatory input from neighboring P cells (cyan) was considerably weaker than the thalamic input (Fig. 2C, middle, cyan vs. gray) largely due to the small unitary EPSPs [31] and low probability of connection between P cells [31], [35]. Within approximately 50 ms after firing onset, only the thalamic inputs remained.

Each synaptic component increased with the number, N_max_, of thalamic inputs (Fig. 2E). The excitatory input from the thalamus (gray) grew nearly linearly from the origin whereas both the inhibitory input from the FS cells and recurrent excitatory input from neighboring P cells appeared only when N_max_ was sufficiently large to cause the neurons to fire (N_max_ ∼20). The FS inhibitory component rose steeply owing to the FS cells' high frequency firing responses to input.

The magnitude of the intracortical synaptic inputs to the P cells also depended on the spatial extent of the thalamic drive. With N_max_ constant, widening the thalamic input from σ = 40 µm (Fig. 2C) to σ = 110 µm (D, middle) activated more FS cells with the result that the inhibition to P cells population increased (compare to Fig. 2C, middle). As σ increased, FS inhibition (Fig. 2F, red) was initially smaller than but then exceeded thalamic excitation (gray). The P cells still fired, albeit more transiently, because the inhibition was delayed with respect to excitation (data not shown). There was only a modest increase in the recurrent excitatory input (cyan).

For the remainder, analyses will be restricted to the first 50 ms after the stimulus onset. The firing rates and the changes in the conductances were greatest in this interval. In addition, *in vivo* recordings from auditory cortex using brief tone pips show that neuronal firing is dominated by thalamic drive: contributions from reverberatory network activity and other cortical areas are substantially smaller [41]. Finally, inputs from low threshold spiking (LTS) interneurons, another major class of inhibitory cells, are unlikely to contribute significantly to firing. Experiments suggest and simulations confirm (not shown) that the weak, facilitating synaptic drive to LTS from the thalamus and local P neurons do not increase substantially during brief stimuli to affect overall network activity [29], [30], [42], [43], [44]).

### Transition between co-tuning and lateral inhibition

As mentioned above, the spatial profiles of inhibitory and excitatory inputs to P cells in the co-tuned network are comparable whereas in the case of lateral inhibition, the inhibitory input is broader. Lateral inhibition and co-tuning could represent separate circuits that differ in e.g., the axonal spread of their associated inhibitory neurons and/or the degree of convergence of inputs from the thalamus. The following simulations suggest, however, that the same circuit can produce both configurations, depending only on the spatial width of the thalamic inputs.

**Figure 3:**
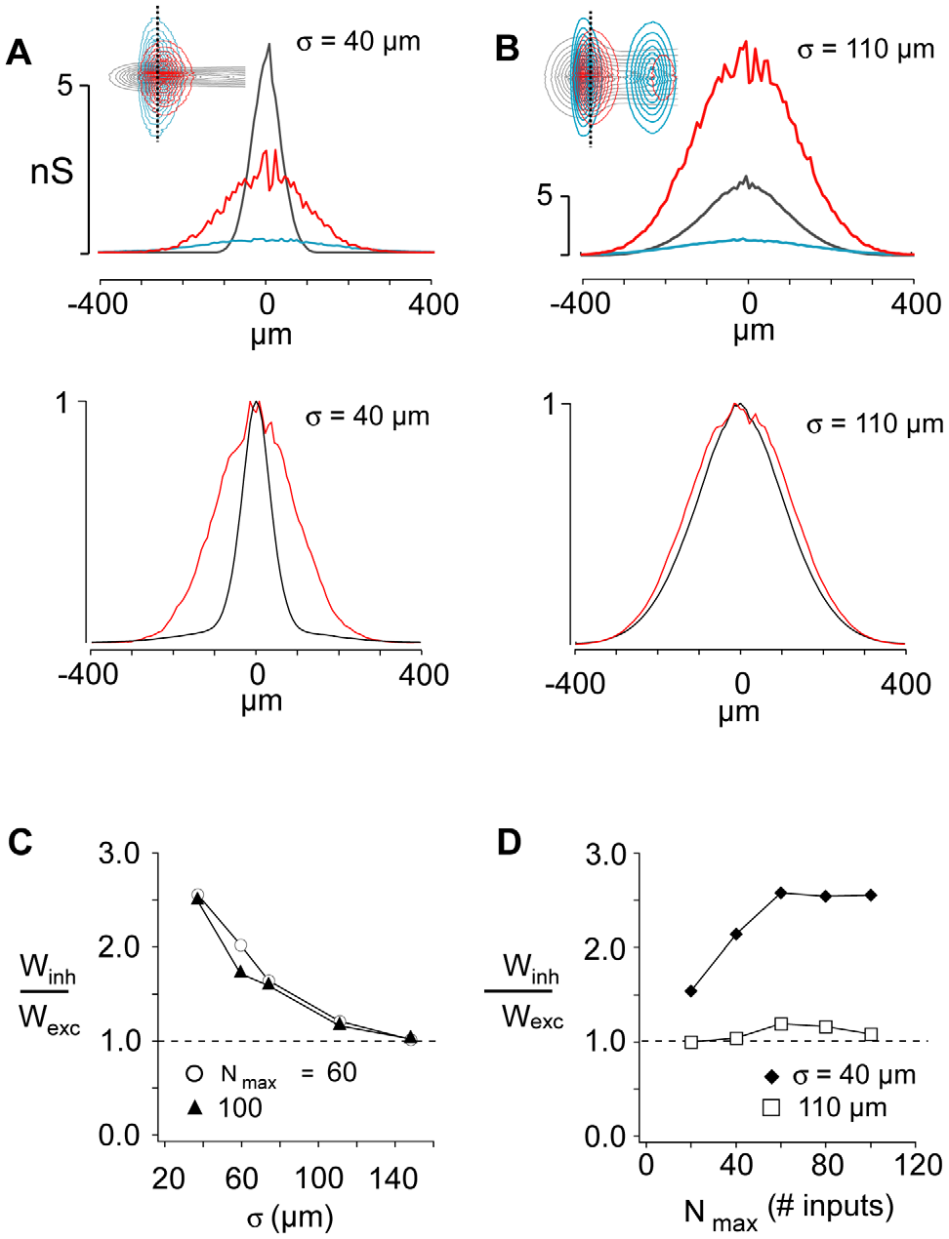
Spatial profiles of synaptic components. **A**, Top, spatial profiles of thalamic (gray), recurrent excitatory (cyan), and FS inhibitory (red) conductances evoked in P cells at different radial distances (abscissa) from the center for σ = 40 µm. Dotted line in inset shows where on the contour plots the spatial profiles were obtained. Bottom, normalized profiles for composite excitatory (black, thalamic + recurrent) and inhibitory (red) conductances generated in P cells **B**, Similar profiles for σ = 110 µm **C**, ratio of inhibitory to excitatory spatial profile half-widths, w_inh_/w_exc_, vs σ for N_max_ = 60 and 100. **D**, w_inh_/w_exc_ vs. N_max_ for σ = 40 µm and 110 µm.

Lateral inhibition predominated when the spatial distribution of thalamic input was narrow. For σ = 40 µm (Fig. 3A, top), both the inhibitory (red) and recurrent excitatory (cyan) inputs were broader than the thalamic input. When combined and normalized (3A, bottom), the excitatory inputs (thalamic + recurrent, black) are substantially narrower than the inhibitory inputs (red), consistent with lateral inhibition. The recurrent P input (Fig. 3A, cyan) was small and contributed only to the foot of the total excitatory distribution.

In the same network, co-tuning was generated when the thalamic input was broad. When σ was increased to 110 µm, the distribution of both inhibitory and recurrent excitatory inputs widened (Fig. 3B, top). However, the rate of widening was proportionately less than the change in thalamic spread (see below for mechanism). As a result, the width of inhibitory profile became nearly equal to that of the composite excitatory width (Fig. 3B, bottom), consistent with the co-tuned configuration.

A plot of the ratio of the inhibitory to excitatory profile widths (W_inh_/W_exc_, measured at half the peak; circles) shows that the network shifted progressively from the lateral inhibitory configuration (W_inh_/W_exc_ >1) to the co-tuned configuration (W_inh_/W_exc_ ∼1) (Fig. 3C). These configurations generally changed very little with N_max_ (Fig. 3D), except for small values where inhibition was not fully activated.

When the network was co-tuned, the balance between excitatory and inhibitory inputs was maintained for neurons at different distances from the center of the network where the thalamic input peaked. The proportions of synaptic conductances of cells in the periphery (Fig. 2D, bottom) were similar to those of cells in the center (middle). When lateral inhibition was predominant, the excitatory-inhibitory balance shifted so that inhibition dominated in cells at the periphery (Fig 2C, compare bottom to middle); for the most distant neurons, only inhibition was present.

### Robustness of simulations

The input dependent transition between lateral inhibition and co-tuning was robust under *in vivo* -like conditions. To simulate background synaptic barrages, white noise current was injected into each P and FS neuron to produce membrane fluctuations with a standard deviation of up to +/− 8mV, similar to what has been observed *in vivo* [45], [46]. The transition between lateral inhibition and co-tuning still occurred, though the region of lateral inhibition was increased slightly (Fig. S1). Background firing would also cause tonic depression of the synaptic potentials, the degree of which differs between thalP, thalFS, PP, PFS, and FSP connections. However, performing the simulations with the calculated steady-state values for the depression at different background firing rates [36], [47] preserved the transition (Fig. S2). The transition also did not depend on the exact details of the connectivity scheme, because simulations using a rate-based model (see below) with a number of non-Gaussian connectivity profiles gave comparable results (Fig. S6).

It should be noted that inhibition must be stronger than the recurrent excitation, as is the case for auditory cortex [33]. The recurrent excitatory input has a spatial profile that is similar in width to that of the inhibitory input (Fig. 3A). Hence, if the amplitudes are comparable, excitation and inhibition cancel in the periphery and lateral inhibition is not possible (data not shown).

Missing from the simulations are the inhibitory connections and electrical coupling between FS cells, both of which have not yet been fully characterized. Mutual inhibition among FS cells would be expected to reduce their firing and hence decrease the net inhibitory input to P cells. To mimic these effects, simulations were performed where the threshold of the FS cells was set at −37 mV, which is 10 mV above the control (Fig. S3). Though firing was reduced by 50%, the shift between lateral inhibition and co-tuning still occurred. The electric coupling between FS cells is likely to have complex effects on the timing of action potentials [48], [49], [50] and cannot be readily predicted without more information about the patterns of connections and coupling strengths. Extensive analyses and simulations are needed to fully characterize the effects, which are beyond the scope of this paper.

### Firing in the co-tuned and lateral inhibitory configurations

**Figure 4:**
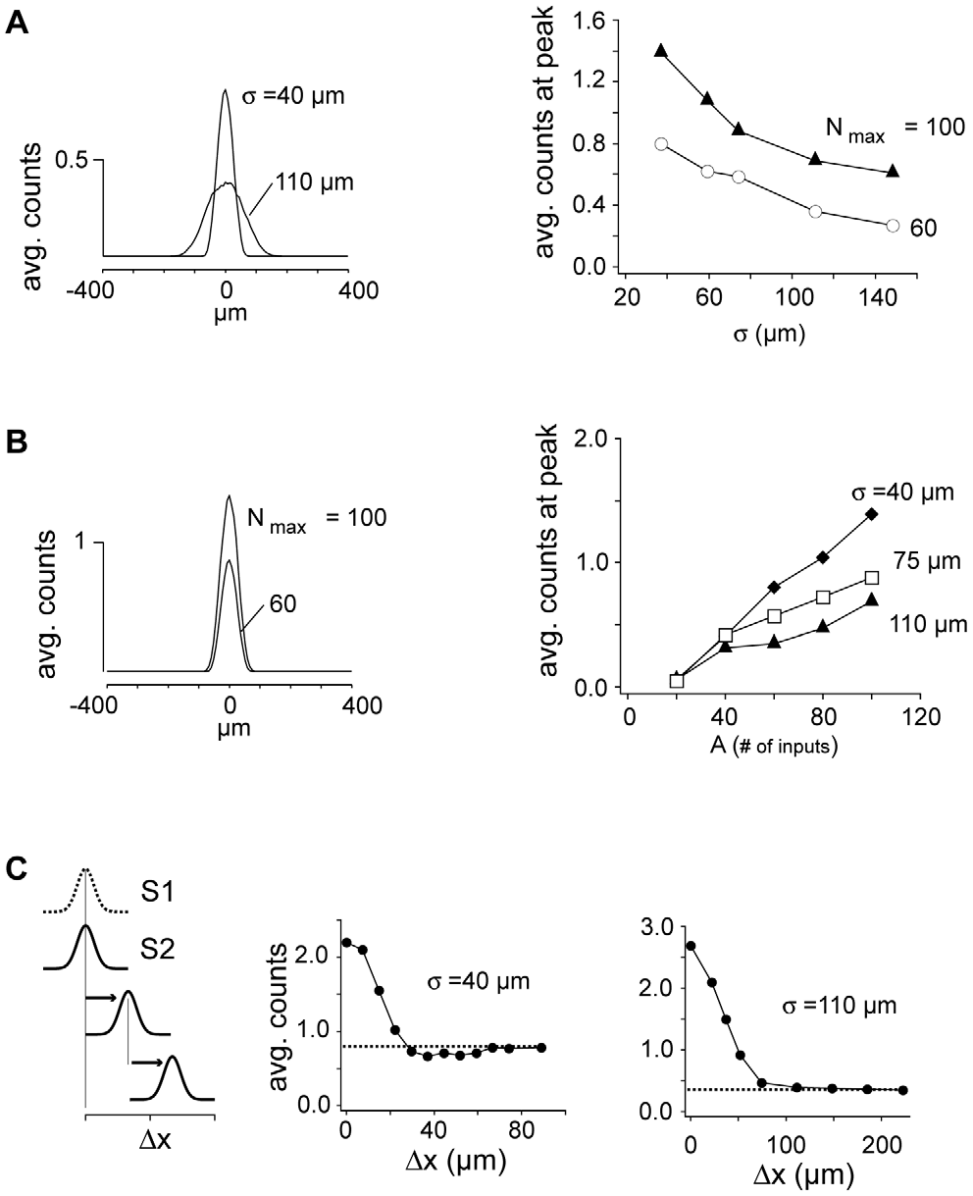
Firing responses of P cells. **A**, left, Average number of action potentials evoked in the first 50 ms of stimulus of cells at different radial distances from the center (abscissa) for broad (σ  = 110 µm) and narrow (σ  = 40 µm) thalamic inputs (N_max_  = 60). Right, plot of counts at the peak of the profiles vs σ for N_max_ = 60 and 100. **B**, left, spatial profile of average counts for N_max_ = 60 and 100 with σ fixed at 40 µm. Right, plot of peak counts vs N_max_ for σ  = 40, 75 and 110 µm. **C**, left, networks stimulated with 2 inputs (S1 & S2), with S2 at different positions (Δx) in the network. N_max_ = 60. Middle, average counts evoked vs Δx for narrow input (σ = 40 µm). Right, average counts vs Δx for broad input (σ = 110 µm).

Firing, quantified as the average counts in a 50 ms interval, was greatest in the lateral inhibitory configuration (Fig. 4A, left). The spatially narrow thalamic input (σ = 40 µm) recruited few FS cells such that the net inhibition was small (Fig. 2C). As the input width increased to approach the co-tuned state (σσ = 110 µm), the firing decreased (Fig. 4A, left) due to increased inhibition (Fig. 2D,F). Peak firing, which corresponds to that of the center cell, decreased systematically with σσ (Fig. 4A, right).

The firing sensitivity of neurons to input was correlated with the extent of lateral inhibition. Increasing the number, N_max_, of thalamic inputs from 60 to 100 evoked more firing, with a modest change in the overall width of the profiles (Fig. 4B, left). To a first approximation, firing increased nearly linearly with N_max_ (Fig. 4B, right). The slope of the relation was steep when σσ was small (40 µm) but became shallower with increasing σσ (75, 110 µm). Note that the slope change was not accompanied by horizontal shifts in the curves, consistent with a pure divisive gain change [51], [52]. This modulation of sensitivity by σσ is a novel form of gain control.

To examine the interaction of multiple inputs, two Gaussian stimuli (S_1_(x), S_2_(x)) separated by different distances (Δx, Fig. 4C left) were delivered to the network. This simulates e.g., two-tone stimuli, which produces side-band suppression of firing rate [18], [19]. The positions of the Gaussians represent the tone frequencies along the tonotopic axis [9], [10], [53]. In the lateral inhibitory configuration (middle), the evoked firing was greatest at Δx = 0 and decreased with Δx to a level below that evoked with a single stimulus (dashed line). When the network was co-tuned, the firing decreased monotonically with Δx, eventually converging with single stimulus firing (right).

### Basis for the transition between co-tuning and lateral inhibition

**Figure 5:**
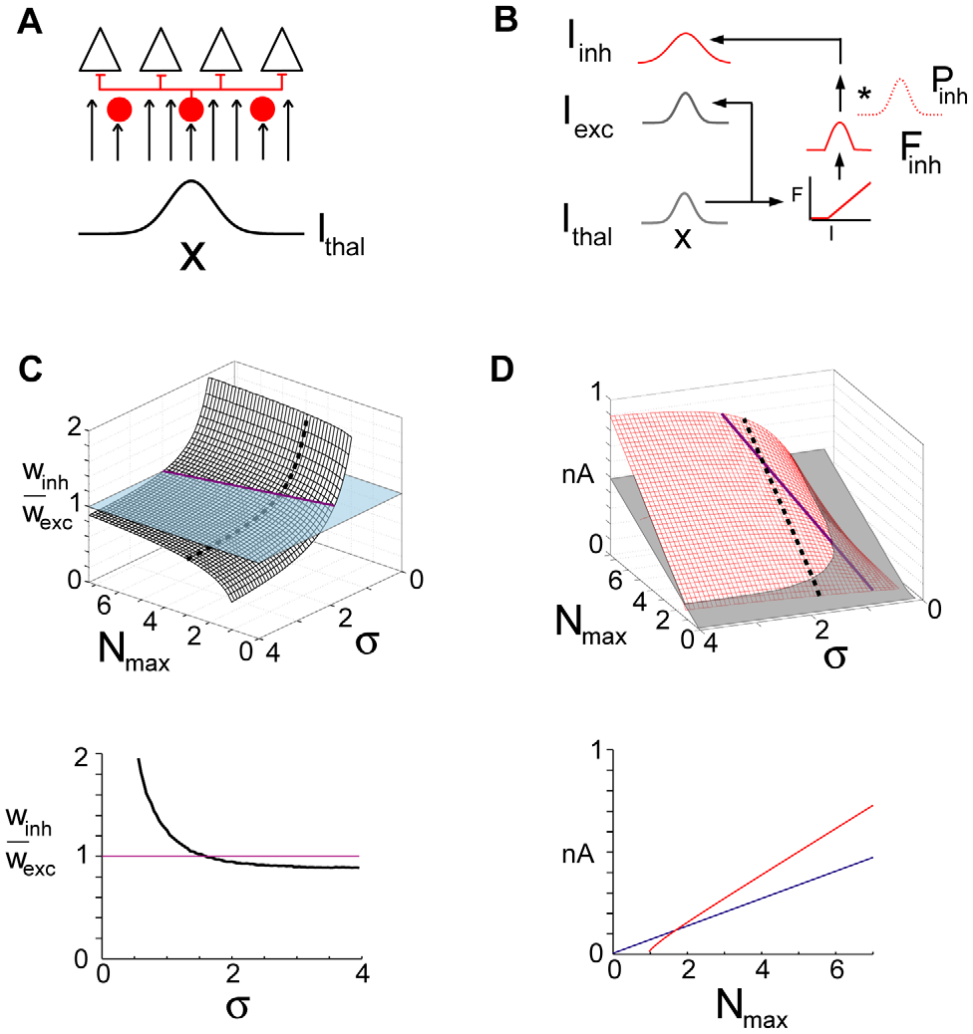
Activity in the feedforward network. A , Schematic of network architecture. Inhibitory cells innervate excitatory cells; both receive spatially distributed inputs (*I_thal_*) from a presynaptic population of cells. **B**, Procedure for calculating spatial profiles of excitatory (*I_exc_*) and inhibitory (*I_inh_*) inputs to excitatory cells. **C**, top, ratio of *I_inh_* to *I_exc_* widths, w_inh_/w_exc_, as a function of N_max_ and σ. N_max_ is normalized (divided) by the minimum current needed to evoke excitatory cell firing and σ is normalized by the standard deviation of the inhibitory spread (σ*_inh_* of *P_inh_* in **B**). Intersection with unitary plane (purple) gives values of N_max_ and σ where *I_exc_* and *I_inh_* are perfectly co-tuned. Bottom, plot of w_inh_/w_exc_ vs σ at N_max_ = 3 (corresponding to dotted line at Top). **D**, Peak excitatory (gray) and inhibitory (red) current plotted against N_max_ and σ. Purple line corresponds to values of N_max_ and σ that produced perfect co-tuning in C. Bottom, plot of peak excitatory (blue) and inhibitory (red) vs N_max_ for σ = 1.6 (corresponding to dotted line at top).

The mechanism underlying the shift between co-tuning and lateral inhibition can be understood by using a reduced model to examine the shifts in excitatory and inhibitory balance. The PFS connections and the weak recurrent excitatory inputs from local P cells were omitted. Under these conditions, the network reduced to a feedforward network where the thalamic afferents synapsed onto both excitatory and inhibitory cells, with the inhibitory cells in turn synapsing onto the excitatory cells (Fig 5A). For the following, the presynaptic thalamic synaptic current *I_thal_* was Gaussian distributed in space and was parameterized by the peak current, *I_max_*, and spatial spread σ (Fig 5B) as in the above simulations.

The spatial profile of the excitatory inputs (*I_exc_*) to both excitatory and inhibitory cells was inherited directly from, and was therefore identical to, *I_thal_* (Fig. 5B). On the other hand, generation of the inhibitory input profile (*I_inh_*) to excitatory cells involved several steps since the inhibitory cells must be activated. First, *I_thal_* was transformed with a threshold-linear function (Methods, equation 6; analogous to firing rate – current relations) to obtain the spatial profile of inhibitory cell firing rate, *F_inh_*,. The presence of the threshold precluded recruitment of weakly driven inhibitory cells far from the center so that *F_inh_*,. was narrower than *I_exc_* (the so-called ‘iceberg’ effect, c.f. [13]). Second, to account for the axonal spread of inhibitory cells to neighboring excitatory cells [54], *F_inh_* was convolved with *P_inh_*, the connection probability profile between inhibitory and excitatory cells (Fig. 1A, right). Finally, multiplying by a constant that has units of nA/Hz gave *I_inh_*.

The network shifted between co-tuning and lateral inhibition as the spatial profile (N_max_ and σ) of *I_thal_* changed. Plotting the ratio of the widths of *I_exc_* and *I_inh_* (*w_inh_/w_exc_*) as a function of N_max_ (normalized by rheobase current) and σ (normalized by the standard deviation, σ *_inh_*, of *P_inh_*) revealed the regimes (Fig. 5C) observed in the full network **(** Fig. 2C,D). When σ was small, *w_inh_* was broader than *w_exc_*, indicating the lateral inhibitory configuration. As σ increased, *w_inh_/w_exc_* reached a regime where *I_exc_* and *I_inh_* were perfectly co-tuned (intersection of the surface with unitary plane) followed by a regime where *I_exc_* was slightly wider than *I_inh_*. With increasing N_max_ (here, N_max_ is the maximum synaptic current from the thalamus), the ratio was initially less than one but grew as the inhibitory cells became more active. Taking a slice through the *w_inh_/w_exc_* surface (dotted line in Fig. 5C, top) reproduces qualitatively the plot in Fig 3C. The model also predicted the relative changes in the peak excitatory and inhibitory current magnitudes (Fig. 5D) observed in the full network simulations. A slice through the surface (dotted line) reproduces qualitatively the plot in Fig. 2E.

The transition still occurred in absence of the I cell threshold (Fig. S4). However, the presence of the threshold minimized the input σ needed for the transition. Without the threshold, even cells far from the center were activated so that the width of *F_inh_* is equal to *I_thal_*. After the convolution with the axonal spread, *w_I_/w_E_* asymptoted toward but was always >1 (Fig. S4). Larger values of σσ were needed to achieve the near-co-tuned regime.

For simplicity, the analyses assumed that the thalamic afferents were distributed equally to excitatory and inhibitory cells. Whether or not the P and FS cells have the same tuning properties in intact animals is unclear. To determine the effects of unequal tuning, the relative widths of thalamic inputs to P and FS were varied (Fig. S5). The shifts still occurred though a broader (narrower) input to inhibitory cells shifted the location of exact co-tuning (*w_inh_/w_exc_*  = 1) toward larger (smaller) σ values.

### Dependence of firing rates on N_max_ and σ

**Figure 6:**
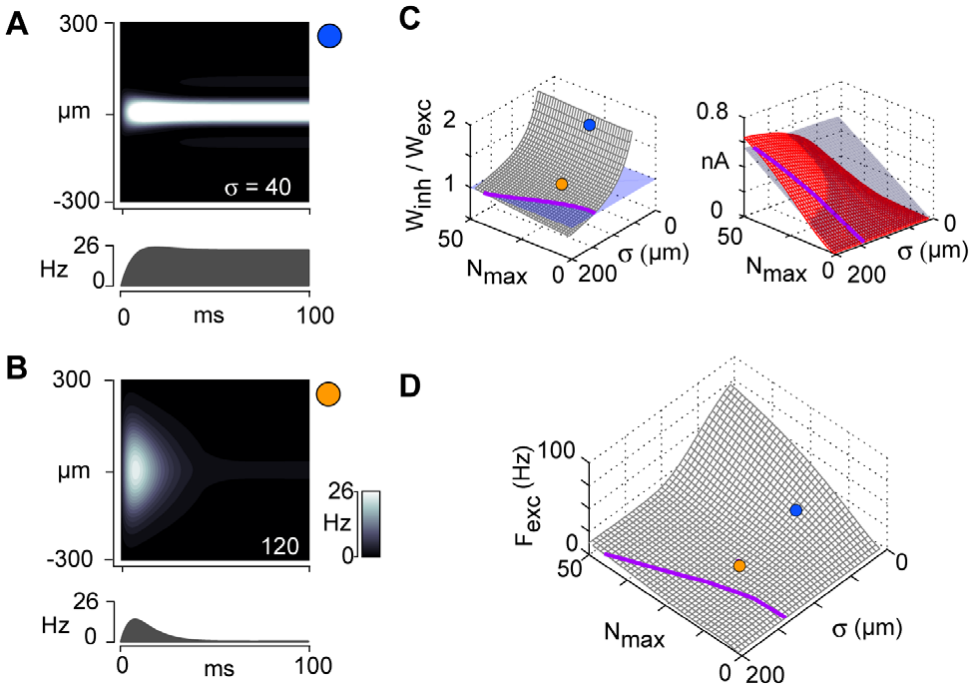
Variation of firing with input. Rate models were used to calculate the firing in the σ σ – N_max_ space (see Methods). The network had a single spatial dimension with connection profiles derived from *in vitro* data. **A**, **B,** spatiotemporal pattern of excitatory cell firing rates for narrow input (σ = 40 µm, A and broad input (σ = 120 µm, B). N_max_ = 20 for both panels. **C**, left, ratio of widths of inhibitory to excitatory current input to excitatory cells vs. N_max_ and σ. Purple line denotes co-tuning. Blue and yellow circles correspond to values of N_max_ and σ used in A and B, respectively. Right, peak excitatory (gray) and inhibitory (red) current (c.f. Fig. 5D). Purple line denotes perfect co-tuning. **D**, mean firing rate (calculated over the first 50 ms) for the center excitatory cell vs. N_max_ and σ.

To determine the changes in firing rate associated with the changes in network configuration, we performed simulations using a rate-based model [55], [56]. Input was a constant current with a Gaussian spatial profile parameterized by N_max_ (maximum thalamic current) and σ. In the LIN regime (σ = 40 µm), firing was confined to a narrow band (Fig. 6A, top) and was sustained (bottom; firing rate profile shown for neuron in center). With relatively broad input (σ = 120), the spatial profile of firing rapidly narrowed within 50 ms (Fig. 6B, top), reflecting the fact that firing was transient (bottom). As above (Fig 3C,D; Fig. 5C,D), increasing the input width (σ) shifted the network from lateral inhibition toward co-tuning (Fig. 6C, left) and produced an associated increase in the magnitude of inhibition (right).

**Figure 7:**
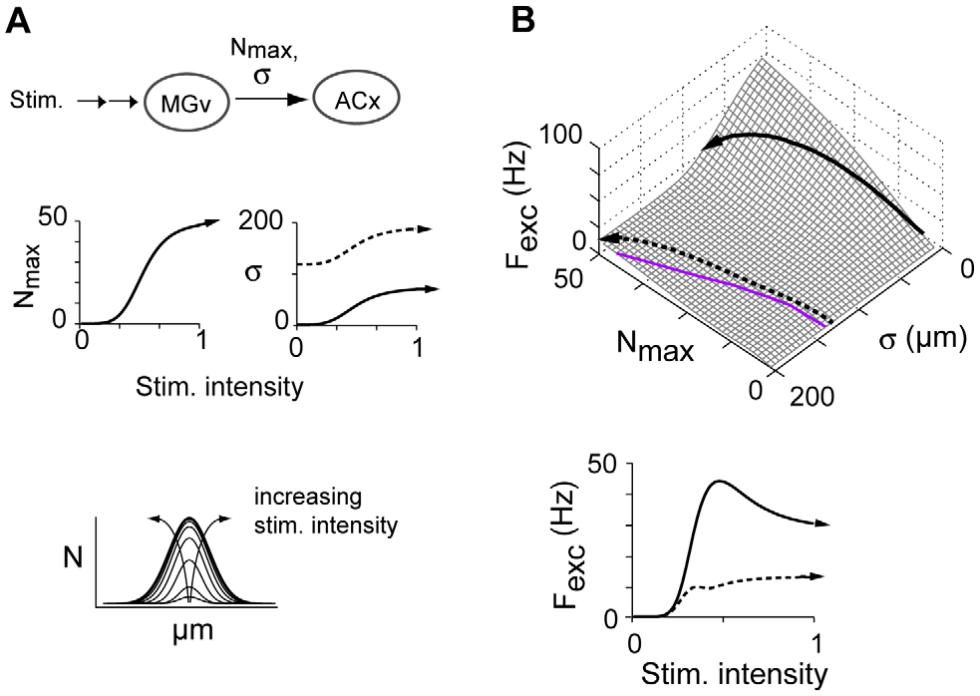
Response trajectories in the σ– N_max_ space. **A**, top, input to auditory thalamus (MGv) is relayed to cortex as a Gaussian activity profile parameterized by N_max_ and σ. Middle, transfer functions for specific stimulus-response trajectories. Curves were Naka-Rushton functions (eqn 9) with parameter values: M = 1000, n = 5, θ = 0.4 (N_max_); M = 75, n = 4, θ = 0.5 (σ); the curve for broad σ (dashed) was additionally shifted up by 75 µm. A single graded stimulus generates concurrent increases in N_max_ and σ of the Gaussian input profiles (schematized at bottom). **B**, top, excitatory cell firing rate (F_exc_) vs. N_max_ and σ. Solid and dashed curves correspond to the transfer functions in A, middle. Gray line denotes perfect co-tuning (c.f. Fig. 6B,C). Bottom, F_exc_ vs. stimulus intensity.

To document the changes in firing with stimuli, the average rate (calculated over the first 50 ms of the firing profiles of the center neurons, Figs. 6A,B, bottom) is plotted against N_max_ and σ (Fig. 6D). As expected, firing was greatest when lateral inhibition predominated, and decreased for increasing σ and decreasing N_max_. This surface essentially describes the change in firing behavior as the input to the network changes. Physiologically, N_max_ and σ of the Gaussian may represent the change in e.g. thalamic input to primary auditory cortex as the stimulus intensity increases (Fig. 7A, top).

To illustrate, we use sigmoid functions to simulate the changes in N_max_ (Fig. 7A, middle left) and σ (right) that may occur with graded increases in stimulus intensity [8]. The resultant Gaussians become taller and wider as intensity increases (bottom). The solid and dotted curves in the σ vs. intensity plot (Fig. 7A, middle, right) represent two scenarios where thalamocortical afferent spreads are relatively narrow (solid curve) and broad (dotted curve), respectively [57], [58].

The changes in σ and N_max_ with intensity resulted in the trajectories shown by the curves traversing the average firing rate surface (Fig. 7B). When the input spread was narrow (solid black curve, Fig. 7B, top), the peak firing rate climbed the steep region of the response contour before rolling off toward the end, resulting in a non-monotonic stimulus-response curve (bottom, solid; c.f. [3], [4]). With broader input (dotted curve), the trajectory was shifted to the flatter part of the contour, yielding a monotonically increasing response (bottom, dotted). Thus, monotonic and non-monotonic rate intensity functions were obtained when co-tuning and lateral inhibition, respectively, were predominant, as predicted previously [8] and observed experimentally [59].

## Discussion

Neuronal firing depends substantially on the interaction between excitatory and inhibitory neurons. Previously, we showed that both co-tuning and lateral inhibition may be needed to account for the diverse firing properties observed in auditory cortex and possibly in other sensory cortices as well [8]. The neuronal architecture underlying both and whether or not they could co-exist is unclear. Using network simulations based on the connectivity data, we demonstrate here that the same network can generate both co-tuning and lateral inhibition, depending only on the spatial distribution of the input. As discussed below, switching between lateral inhibition and co-tuning may be realized under physiological conditions in two ways: distinct sets of afferents with different axonal arborizations may innervate a given cortical area or alternatively, the spatial spread of inputs to a cortical area may be modulated by the stimulus.

The firing responses of neurons were markedly different in the lateral inhibitory versus co-tuned configurations and reproduced the heterogeneous response properties and receptive fields observed in A1 with acoustic stimuli [1], [2], [3], [4], [19]. Depending on whether lateral inhibition or co-tuning predominates, firing may be phasic or tonic (Fig. 6), may change monotonically or non-monotonically with intensity (Fig. 7), and may or may not exhibit sideband suppression (Fig. 4). Thus, the diverse response range observed physiologically may stem from a single network architecture.

### Functional implications

The results of the simulations are robust under a variety of conditions (see Supporting Text S1). The main observation is maintained with different models (Fig. 3 vs Fig. 5 vs. Fig. 7) and with different network and input parameters (Figs. S1-S3, S5). Nevertheless, several caveats must be considered. One is that the model applies only to cortical regions where graded changes in response properties reflect the orderly arrangement of thalamic afferents and FS cell arbors. These conditions appear to be met in rodent A1; frequency tuning of neurons in the middle layers of A1 has been found to vary systematically, with substantial changes in tuning over a few hundreds of µm on the rostrocaudal axis, and thalamic afferents showed a comparable degree of tonotopic organization in a recent study [60]; but see [11]. Likewise the model could potentially apply to phenomena such as the graded changes in orientation tuning within a single ocular dominance column of primary visual cortex (V1 [61]).

Another proviso is that the FS-mediated inhibition must be stronger than the recurrent, intracortical excitation. This condition is met in auditory and somatosensory cortices where connection probabilities and the unitary synaptic potentials are much larger for FS to P than for P to P connections [33], [52], [62], [63]. Prominent inhibitory synaptic conductances are also evoked *in vivo* during auditory stimulation [13], [14], [64]. In primary visual cortex, the role of inhibition in shaping the tuning of cells remains controversial [20], [65]: strong transient inhibition has been evoked by electrical stimulation of the thalamus [66] though generally not with visual stimuli [67], [68], [69]; but see [70].

In addition, the model while incorporating many of the measured parameters is necessarily incomplete because several parameters - notably the spatiotemporal profile of thalamic inputs to P and FS, and sources of noise under *in vivo* conditions - are yet poorly characterized. Nevertheless, simulations where these parameters are varied yield qualitatively, if not quantitatively, similar results (Figs. S1 **–**S5). The simulations also do not consider other types of interneurons and potentially important inputs from other cortical areas. Many of these issues can be circumvented if only the synaptic events and firing within the first 50 ms after the stimulus are considered. In the auditory system, as noted above, brief tone pips (25**–**50 ms) are often used to characterize the tuning and firing properties of neurons. As argued in the Results, the synaptic inputs to P cells would be dominated by excitatory inputs from the thalamus and inhibitory inputs from the FS cells.

Finally, *in vivo* whole-cell recordings from the auditory system of mice and rats [13], [71] have suggested that excitation and inhibition are co-tuned, though there is some evidence that the co-tuning is only approximate [15], [16]. The apparent lack of lateral inhibition may mean that the spatial extents of the thalamic afferents from the medial geniculate are relatively broad so as to bias the network towards co-tuning. Alternatively, the experimental conditions may bias the network towards co-tuning. All of the *in vivo* experiments thus far have been with anesthetized animals. The evoked responses in awake animals are markedly different [5], [72], and are more consistent with the presence of lateral inhibition [8]. Conservatively, our findings are most comparable to the *in vivo* studies in anaesthetized animals using brief stimuli. However, the transition between lateral inhibition and co-tuning persists in the presence of background noise (Fig. S1) and synaptic adaptation (Fig. S2), more similar to conditions that obtain in the awake state or with prolonged and/or natural stimuli.

With these caveats, the results of the simulations have several implications. First, because the recurrent excitatory connections are weak, the firing of P cells is determined primarily by a feedforward configuration (thalamic to P, FS; FS to P). There is some experimental support for this finding in auditory cortex *in vivo*, because blocking recurrent excitation was found not to grossly affect the tuning of neurons responding to brief stimuli [41]. During prolonged stimuli, there may be a greater contribution from recurrent excitation; non-FS cells may also play a larger role with prolonged stimuli, because physiological studies have shown that PSPs to and from some non-FS cells facilitate [29], [30], [42], in contrast to the depressing synapses between P and FS cells.

A second implication of the model is that the magnitude of inhibition increases in parallel with the spread of thalamic input (Fig. 5D and 6C). The resultant decrease in excitatory cell firing (Fig. 4A) resembles that observed when auditory stimuli become more broadband [59] or when the size of visual stimuli expands beyond the classical receptive field [73], [74], [75], [76].

A third implication is that the thalamocortical terminal field widths will determine whether a cortical area is biased towards co-tuning or lateral inhibition. In rodent somatosensory and primate visual cortices, the spatial distribution of thalamic axons varies 6 fold in the thalamorecipient layers [57], [58]. In auditory cortex there are few reports on the widths of thalamocortical terminal fields, and none to our knowledge in rodents. However, frequency layer organization in rat [77] and gerbil [78] auditory thalamus shifts from narrow at the caudal end to broad at the rostral end; similar shifts in laminar organization are observed in the cat [79], [80]. These thalamic pattern differences have been postulated to underlie heterogeneous response properties of neurons within an isofrequency band [59]; c.f. [77], [81], [82], [83]. The model predicts that if the distinct sets of inputs differ in their axonal arborization widths, this variation alone is enough to support a wide range of receptive field properties seen experimentally in rodent A1.

Fourth, whether the network is biased toward lateral inhibition or co-tuning will also depend on the dendritic and axonal arborizations of FS cells [62], [84], [85], [86], [87], which may vary along with other properties of the specific sub-circuits targeted by broad vs. narrowly distributed thalamic axons [88]. In somatosensory cortex, the spatial distribution of FS cell processes is well conserved, suggesting systematic shifts in network configuration that parallel the changes in the distribution of thalamic afferents.

Finally, the fact that the relation between excitation and inhibition is malleable in a single network potentially provides a mechanism for modulating the response of the network to a variety of inputs and behavioral states. Recently, for example, the magnitudes of feedforward and lateral inputs in visual cortex were found to be modulated by stimulus contrast [89]. It would be of interest to determine experimentally if the relative degree of co-tuning vs. lateral inhibition can be triggered by changing the stimulus characteristics, experimental conditions, or state of the animal. It will also be interesting to see whether the reduced model presented here can account for response properties obtained with complex sound stimuli in awake animals [2], [5], [72], or whether additional elements such non-FS inhibitory neurons, input from other cortical areas, and state-dependent effects of neuromodulators are important.

## Methods

### Full network model

The network was a 2 dimensional sheet of 160×160 P and 32×32 of inhibitory FS cells (Fig. 1A). The density of neurons was 91125 neurons/mm^3^ and network was assumed to correspond to a volume (x by y by h) of 1185×1185×200 µm before compression to 2 dimensions (1185×1185 µm). In 2 dimensions, the probability that a reference neuron at x_0_, y_0_ is connected to its neighbors x_i_, y_j_ is given by:
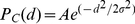
(1)where *d* represents radial distance 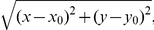 *A* represents peak probability, and σ represents spread of connectivity (see Results for values); fits to the experimental data (not shown) were done using nonlinear-least squares regression. Probability values predicted from the fitted curves did not differ significantly from the experimental values (p>0.05, χ^2^ goodness-of-fit tests).

The neurons were adaptive exponential integrate and fire units (aEIF; [39]; see Supporting Text S1). The aEIF accurately reproduces the firing patterns of cortical neurons with relatively little computational cost, thereby allowing modeling large networks efficiently. The parameters governing the firing behaviors (Table S1) were adjusted so as to produce firing patterns and firing rate-current (F/I curves) that resembled those of the experimentally recorded P and FS cells.

The postsynaptic conductance, g_syn_, was described with an alpha function:



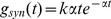
(2)The parameters k and α were adjusted so that the amplitudes and time courses of resultant EPSPs and IPSPs matched experimentally measured synaptic potentials evoked in the two cell types (not shown). Short-term depression and facilitation were implemented using a phenomenological model [36], [47]. For connections between cortical neurons, model parameters (Table S1) were adjusted to match the experimentally measured synaptic dynamics. The amplitude and short-term dynamics of thalamic EPSPs evoked in P and FS neurons were taken from data obtained in mouse thalamocortical slices [40] Schiff and Reyes, unpublished) and obtained from somatosensory thalamocortical slices [43], [44].

The thalamic inputs to the network were drawn randomly from a set of simulated spike trains (Fig. 1B, inset). For each train, the spikes occurred repetitively at a specified rate F; the latency of the first spike was Gaussian distributed (mean = 1/F; standard deviation = 0.25/F) as was the latency of each successive spike. With this procedure, the population spikes tended to cluster at the onset of the stimulus but over time became more evenly distributed as the spikes became less synchronous, resulting in a histogram with a phasic-tonic firing profile (Fig. 2B, bottom). Both the number (N_in_) of inputs and frequency F_in_ of each input to the network were Gaussian distributed in space (Fig. 1B) so that cells in the center received the maximum number of inputs (N_max_ = 25**–**150) firing at the maximum specified rate (F_max_ = 50 Hz):



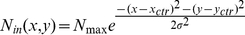
(3)




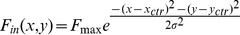
(4)where x_ctr_ and y_ctr_ are the center of the network. N_in_ and F_in_ were adjusted so that the evoked firing rates of the P and FS cells were in the midrange of their respective F/I curves. The spike trains were used to calculate the composite synaptic currents generated in each P and FS neuron. Representative synaptic conductances and associated firing are shown in Figure 1C.

The evoked firing was quantified by calculating the number of spikes that occur within a 50 ms time window after the onset of the stimulus. This corresponded approximately to the peak of the post-stimulus time histogram (PSTH) compiled from the spike trains (Fig. 1D).

### Calculation of excitatory and inhibitory tuning in feedforward networks

The simplified network shown in Figure 5 consisted of inhibitory cells that synapsed onto excitatory cells; both excitatory and inhibitory cells received spatially distributed inputs from the thalamus. The spatial connection profile of the inhibitory to excitatory cells (P_inh_(x)) and that of the thalamic synaptic inputs (I_thal_) to the excitatory and inhibitory cells are each Gaussian:



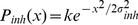
(5a)

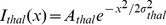
(5b)where σ_inh_ and σ_thal_ are the standard deviations of the inhibitory to excitatory connection profile and the thalamic input, respectively. The spatial profiles of the excitatory (I_exc_) and inhibitory input (I_inh_) were calculated as described in Results. The transformation of thalamic input to obtain inhibitory firing was given by:



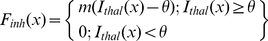
(6)where θ is the threshold current for firing and m is the slope of the firing-current relation. Additional simulation details, including parameter values, are in the Supporting Information.

### Firing rate model

The simulations in Figures 6 and 7 were based on an established model [53], [54]. The model assumes that the cell population is large and firing is random, so that the calculation of individual spike trains and cell-by-cell responses can be replaced by a simplified expression for excitatory and inhibitory population firing rates (*F_exc_, F_inh_,* in Hz) in terms of position (*x*) and time:



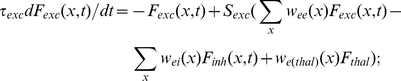
(7a)

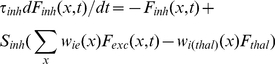
(7b)The time constants *τ_exc,_ τ_inh_* (Table S3) for the population firing rates reflected the relative membrane time constants measured for P and FS *in vitro* (c.f. [8]). The synaptic weight functions *w_ee_, w_ei_, w_ie_* were each the product of three terms:



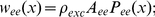
(8a)




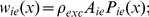
(8b)




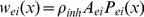
(8c)where *ρ* is presynaptic cell density (Supporting Information, Table S3), *A* is average unitary synapse strength in pA/Hz (table S4), and *P(x)* is the Gaussian connectivity profile (eqn 1, for parameter values, see Results). Thalamic firing rate *F_thal_* was fixed at 20 Hz, while the thalamic input weight functions *w_e(thal)_, w_i(thal)_* were the corresponding average unitary synaptic strengths (Table S4) multiplied by the Gaussian input profiles, parameterized by N_max_ and σ as detailed for Figure 6. N_max_ and σ for thalamic input did not differ between E and FS cells. Because only transient responses were examined, the model did not incorporate short-term synaptic plasticity, which influences network dynamics on longer time scales [36]. The relationship of firing rate to total input current for each cell type (*S_exh_, S_inh_,)*, was modeled as a Naka-Rushton function:



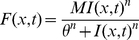
(9)
*M*, *θ*, and *n* (Table S3) were fitted to average plots of non-adapted firing rate versus injected current obtained from cortical P and FS cells *in vitro* (not shown), using an iterative search procedure.

## Supporting Information

Figure S1 **Effects of background noise.** Simulations were performed with the network shown in Fig. 4 of the main text using the same thalamic inputs (number of thalamic inputs Nmax  = 100; σ = 110 µm). White noise current was added to the cell to produce voltage fluctuations in all neurons. **A**, dot rasters showing firing of P (top) and FS (bottom), cell populations. Injected noise amplitude was 1 nA (+/− standard deviation). **B**, Temporal profiles of synaptic conductances to P cells at the center of the network from thalamus (gray), neighboring P cells (cyan), and FS cells (red). **C**, Normalized spatial conductance profiles of composite excitatory (black) and inhibitory inputs (red) to P cell population for σ = 40 (top) and 110 µm (bottom). **D**, ratio of excitatory to inhibitory spatial halfwidths vs s for 3 noise levels.
(TIF)

Figure S2 **Effects of background firing.** Simulations were performed using steady-state values of synaptic depression/facilitation, assuming all neurons were firing spontaneously at different frequencies prior to the arrival of the stimulus. **A**, Spatial profiles of composite excitatory (black) and inhibitory (red) conductances evoked in P cells for input σ of 40 µm (left) and 110 µm (right). **B**, ratio of excitatory to inhibitory spatial halfwidths vs background firing rate for σ = 40 µm (circles) and 110 µm (squares).
(TIF)

Figure S3 **Role of FS excitability in LIN-CON transition.** **A**, simulations with FS cells with lowered threshold (−52 mV). **i**, dot rasters of P cell cells evoked with σ = 40 µm. Bottom shows poststimulus time histgram. **ii**, normalized spatial profile of excitatory (black) and inhibitory conductances evoked in P cells with σ = 40 µm (left) and σ = 110 µm (right). **B**, FS threshold set at −47 mV as in the main text. **C**, FS threshold set at -37 mV.
(TIF)

Figure S4 **Role of FS threshold in LIN-CON transition.** **A**, calculation of spatial profile of excitatory and inhibitory input to P cells is as in Figure 5 of the main text except that the threshold for the inhibitory input was removed so that the transform (F/I curve) is linear. **B**, without the threshold, the surface describing the ratio of excitatory to inhibitory widths approaches 1 asymptotically as σ increases.
(TIF)

Figure S5 **Effects of differences in spatial inputs to excitatory and inhibitory cells.** **A–C**, changes in the ratio of inhibitory to escitatory widths surface as the thalamic input to inhibitory cells was made broader than that to excitatory cells. See Supporting Text S1 for details.
(TIF)

Figure S6 **Transition between lateral inhibition and co-tuning with non-Gaussian connectivity schemes. A,** left, plots of ratio of widths of inhibitory to excitatory current to P cells (W_inh_/W_exc_) versus input width (σ), for N_max_ = 10, 20, or 30, in the rate-based model (c.f. figs. 6 and 7 of main text). Connectivity profiles were uniform (box function, schematized in red, inset). Perfect co-tuning is indicated by dashed line at W_inh_/W_exc_ = 1. Right, spatiotemporal profile of normalized firing rates of P cells for narrow input (σ = 40, top) and broad input (σ = 160, bottom). **B,** corresponding data for connectivity based on a quadratic model; **C**, binomially distributed connectivity.
(TIF)

Table S1 **Parameters of adaptive exponential integrate-and-fire cells.**
(PDF)

Table S2 **Parameters governing dynamic properties of EPSPs and IPSPs.**
(PDF)

Table S3 **Network parameters for the firing rate model.**
(PDF)

Table S4 **Unitary response amplitudes for the firing rate model.**
(PDF)

Text S1 **Additional methods and results.**
(PDF)
