# Supplementary figures and images for: Coexistence of Lateral and Co-Tuned Inhibitory Configurations in Cortical Networks
Source: PLoS Comput Biol. 2011 Oct 6;7(10):e1002161. doi: 10.1371/journal.pcbi.1002161 (PMC3188483; doi:10.1371/journal.pcbi.1002161)

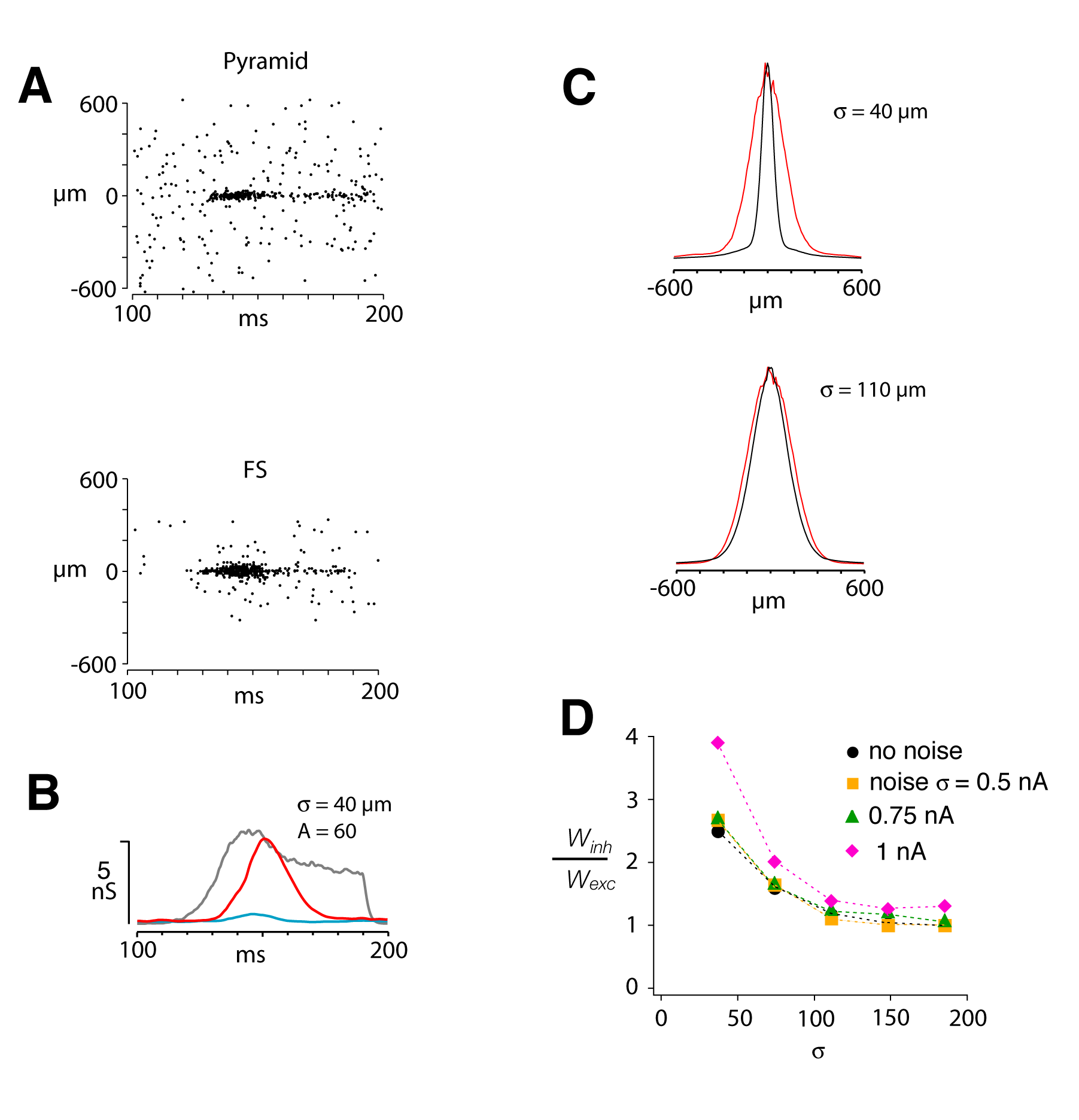

Supplement: Figure S1 — Effects of background noise. Simulations were performed with the network shown in Fig. 4 of the main text using the same thalamic inputs (number of thalamic inputs Nmax = 100; σ = 110 µm). White noise current was added to the cell to produce voltage fluctuations in all neurons. A, dot rasters showing firing of P (top) and FS (bottom), cell populations. Injected noise amplitude was 1 nA (+/− standard deviation). B, Temporal profiles of synaptic conductances to P cells at the center of the network from thalamus (gray), neighboring P cells (cyan), and FS cells (red). C, Normalized spatial conductance profiles of composite excitatory (black) and inhibitory inputs (red) to P cell population for σ = 40 (top) and 110 µm (bottom). D, ratio of excitatory to inhibitory spatial halfwidths vs s for 3 noise levels. (TIF) [file pcbi.1002161.s001.tif]

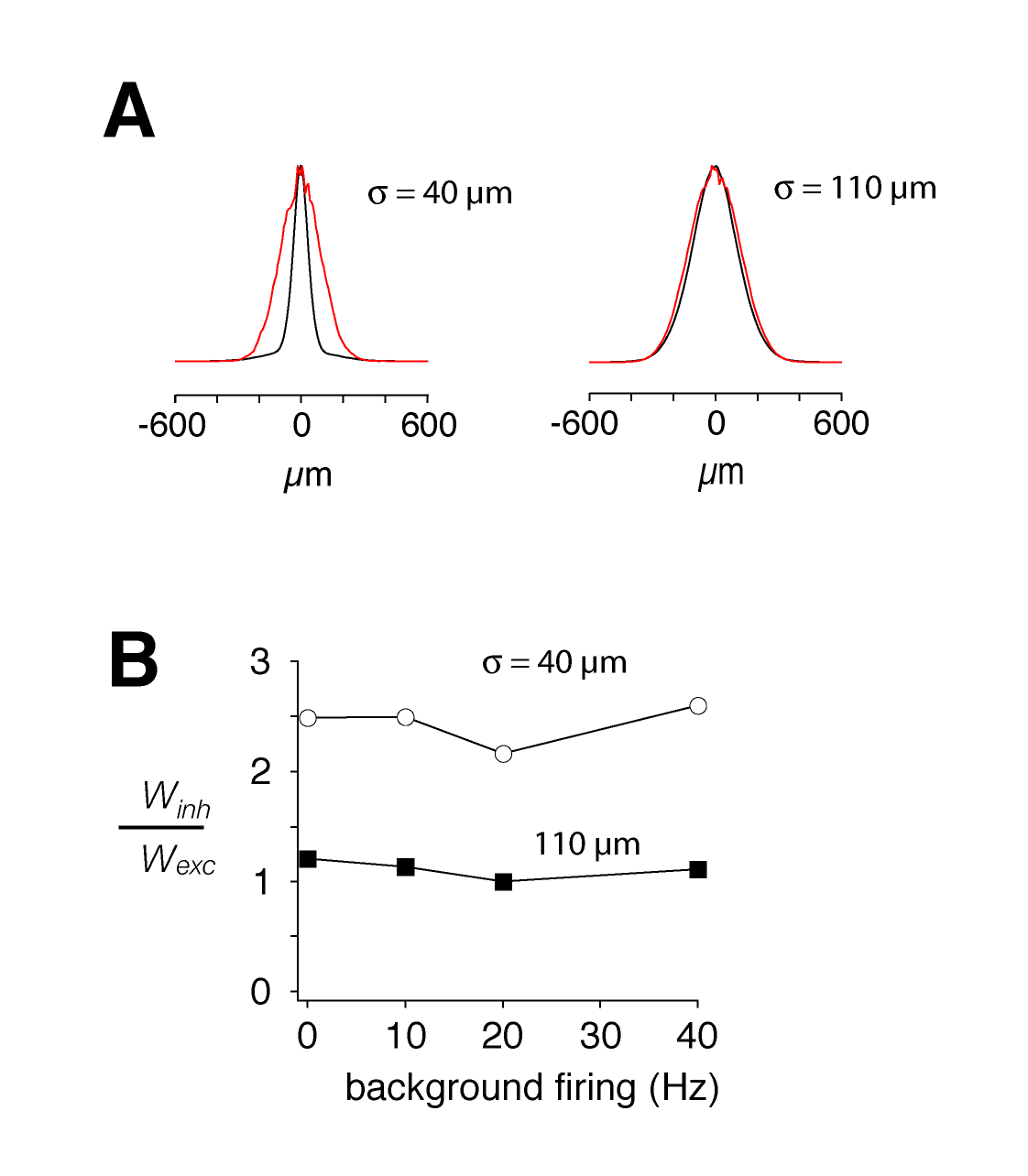

Supplement: Figure S2 — Effects of background firing. Simulations were performed using steady-state values of synaptic depression/facilitation, assuming all neurons were firing spontaneously at different frequencies prior to the arrival of the stimulus. A, Spatial profiles of composite excitatory (black) and inhibitory (red) conductances evoked in P cells for input σ of 40 µm (left) and 110 µm (right). B, ratio of excitatory to inhibitory spatial halfwidths vs background firing rate for σ = 40 µm (circles) and 110 µm (squares). (TIF) [file pcbi.1002161.s002.tif]

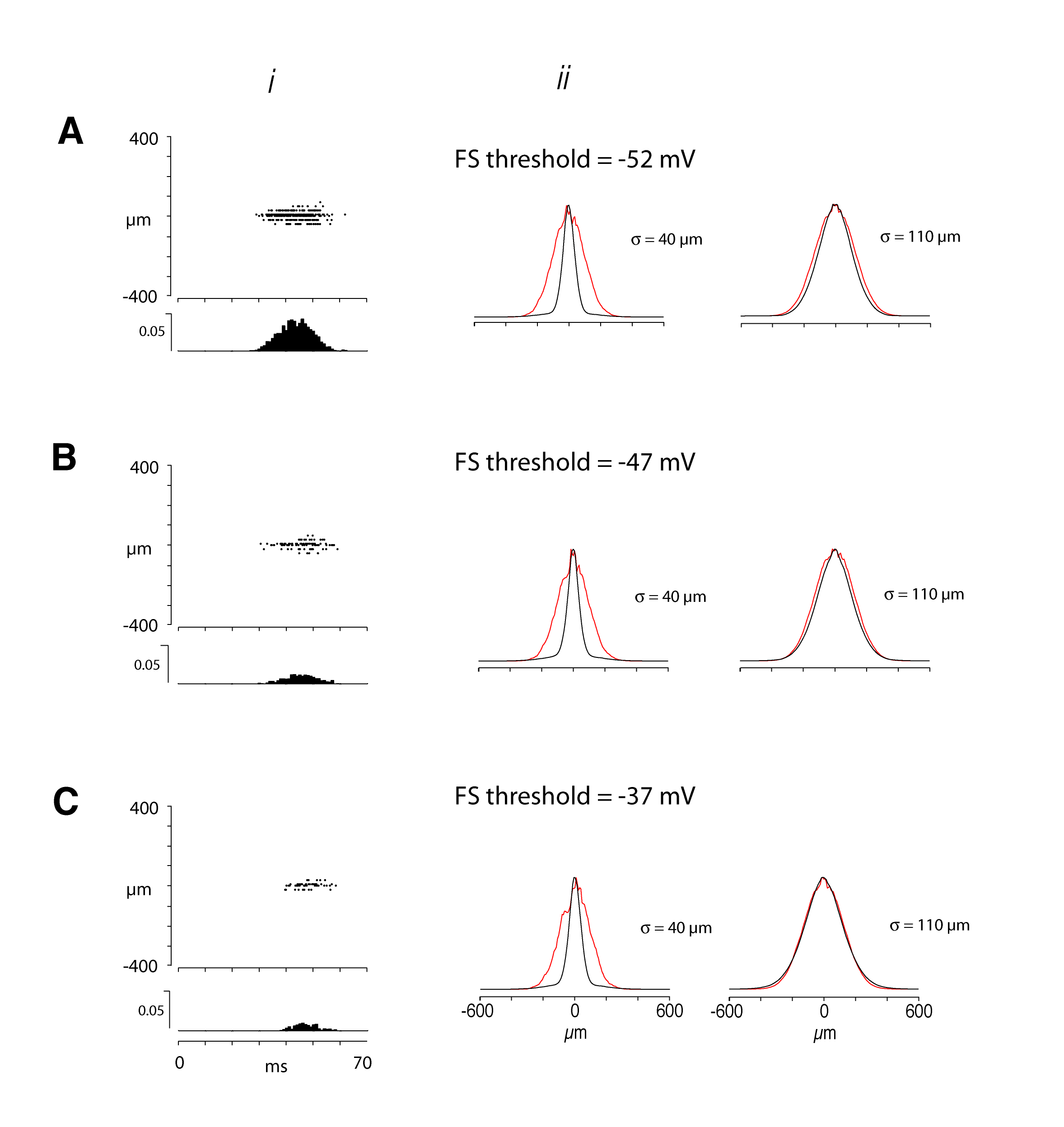

Supplement: Figure S3 — Role of FS excitability in LIN-CON transition. A, simulations with FS cells with lowered threshold (−52 mV). i, dot rasters of P cell cells evoked with σ = 40 µm. Bottom shows poststimulus time histgram. ii, normalized spatial profile of excitatory (black) and inhibitory conductances evoked in P cells with σ = 40 µm (left) and σ = 110 µm (right). B, FS threshold set at −47 mV as in the main text. C, FS threshold set at -37 mV. (TIF) [file pcbi.1002161.s003.tif]

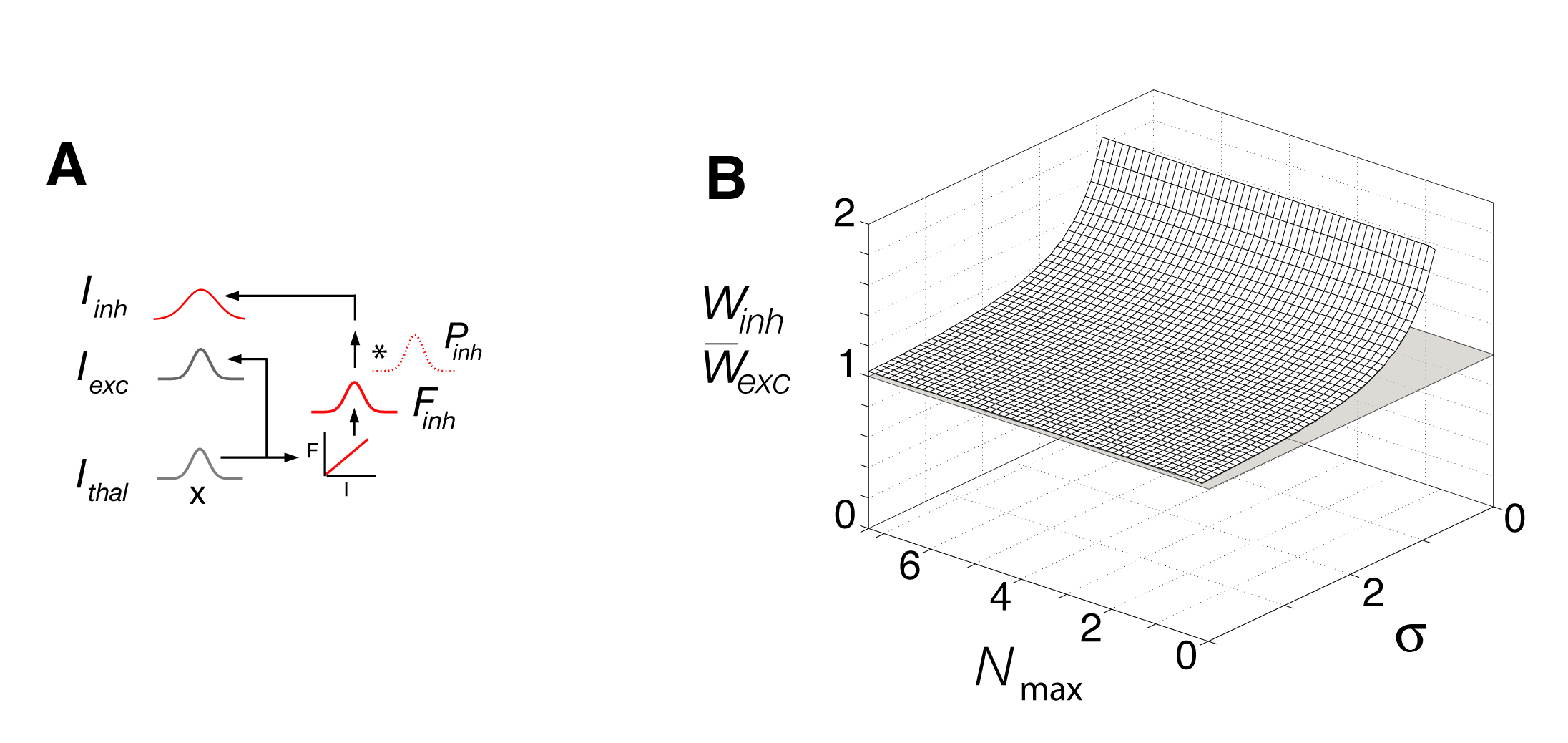

Supplement: Figure S4 — Role of FS threshold in LIN-CON transition. A, calculation of spatial profile of excitatory and inhibitory input to P cells is as in Figure 5 of the main text except that the threshold for the inhibitory input was removed so that the transform (F/I curve) is linear. B, without the threshold, the surface describing the ratio of excitatory to inhibitory widths approaches 1 asymptotically as σ increases. (TIF) [file pcbi.1002161.s004.tif]

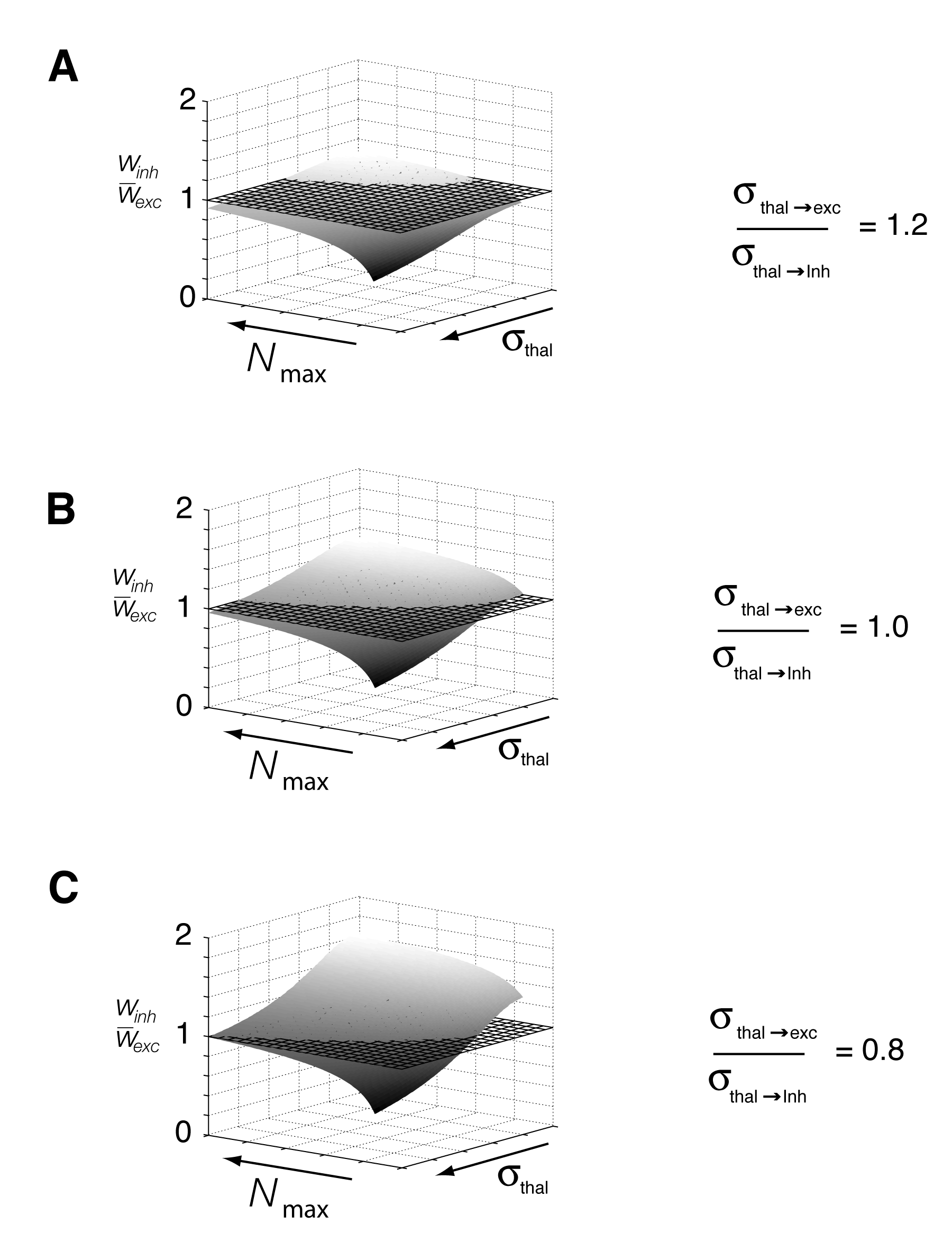

Supplement: Figure S5 — Effects of differences in spatial inputs to excitatory and inhibitory cells. A–C, changes in the ratio of inhibitory to escitatory widths surface as the thalamic input to inhibitory cells was made broader than that to excitatory cells. See Supporting Text S1 for details. (TIF) [file pcbi.1002161.s005.tif]

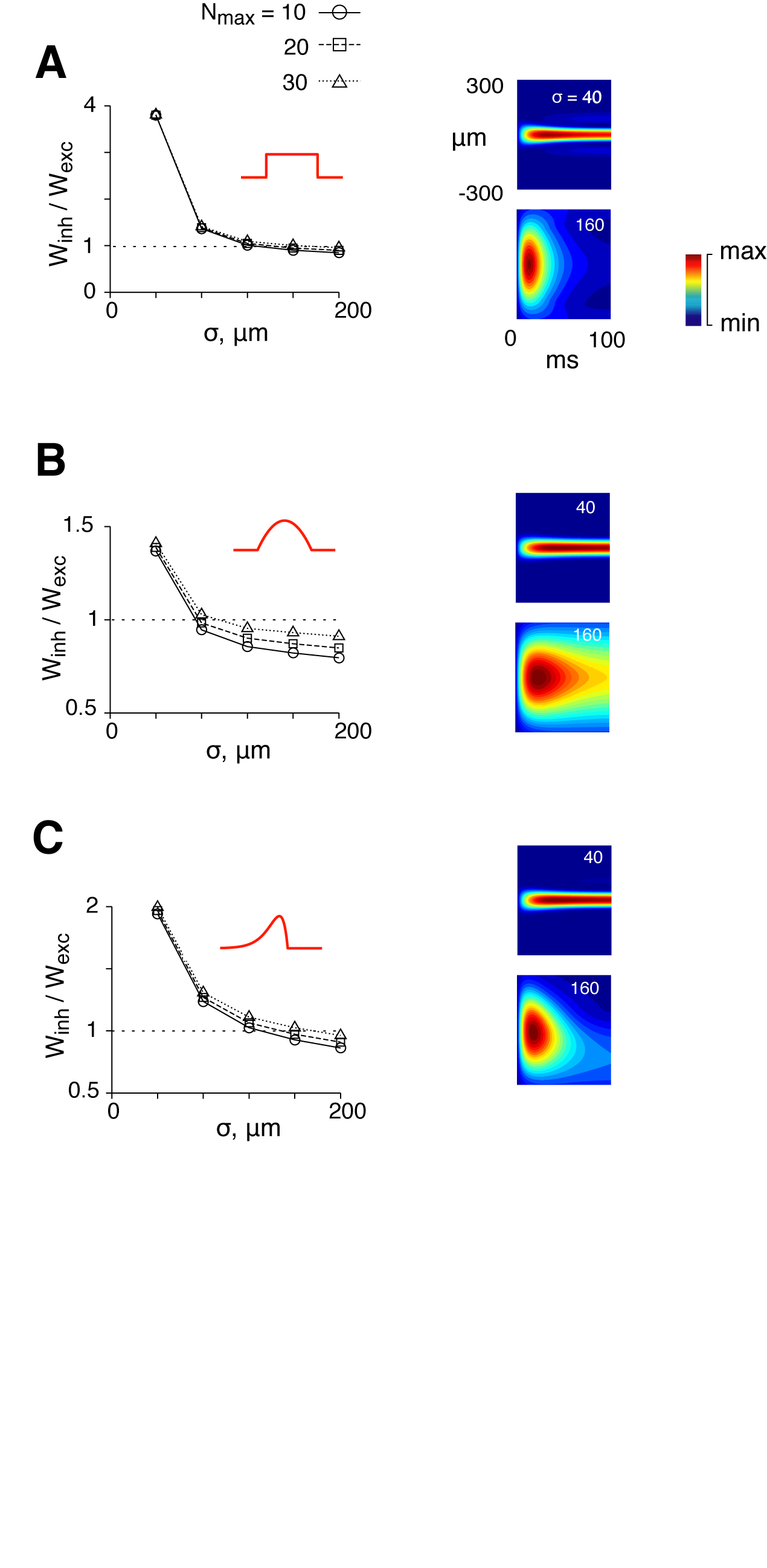

Supplement: Figure S6 — Transition between lateral inhibition and co-tuning with non-Gaussian connectivity schemes. A, left, plots of ratio of widths of inhibitory to excitatory current to P cells (Winh/Wexc) versus input width (σ), for Nmax = 10, 20, or 30, in the rate-based model (c.f. figs. 6 and 7 of main text). Connectivity profiles were uniform (box function, schematized in red, inset). Perfect co-tuning is indicated by dashed line at Winh/Wexc = 1. Right, spatiotemporal profile of normalized firing rates of P cells for narrow input (σ = 40, top) and broad input (σ = 160, bottom). B, corresponding data for connectivity based on a quadratic model; C, binomially distributed connectivity. (TIF) [file pcbi.1002161.s006.tif]
