# Supplementary material for: Coexistence of Lateral and Co-Tuned Inhibitory Configurations in Cortical Networks
Source: PLoS Comput Biol. 2011 Oct 6;7(10):e1002161. doi: 10.1371/journal.pcbi.1002161 (PMC3188483; doi:10.1371/journal.pcbi.1002161)
Supplement: Table S1 — Parameters of adaptive exponential integrate-and-fire cells. (PDF) [file pcbi.1002161.s007.pdf]

Table S1: Parameters of adaptive exponential integrate-and-fire cells

| Cell | $1/g_L$ (MΩ) | $\tau_m$ (ms) | $E_L$ (mV) | $V_T$ (mV) | $V_{reset}$ (mV) | a (nS) | b (pA) | $\Delta_T$ (mV) | $\tau_w$(ms) |
|---|---|---|---|---|---|---|---|---|---|
| P | 182 | 18 | -65 | -45 | -48 | -0.95 | 250 | 0.8 | 60 |
| FS | 119 | 8 | -67 | -47 | -50 | 0 | 80 | 3 | 16 |
