# Supplementary material for: Coexistence of Lateral and Co-Tuned Inhibitory Configurations in Cortical Networks
Source: PLoS Comput Biol. 2011 Oct 6;7(10):e1002161. doi: 10.1371/journal.pcbi.1002161 (PMC3188483; doi:10.1371/journal.pcbi.1002161)
Supplement: Table S2 — Parameters governing dynamic properties of EPSPs and IPSPs. (PDF) [file pcbi.1002161.s008.pdf]

Table S2: Parameters governing dynamic properties of EPSPs and IPSPs

Connection	A (nS)	α	U	τ_{rec} (ms)	τ_{fac} (ms)
P→P	0.145	0.375	0.55	0.45	0
P→FS	0.47	0.55	0.55	0.60	0
FS→P	0.5	0.25	0.555	0.375	0
Thal→P	0.7	0.9	0.25	0.5	0
Thal→FS	1.8	3	0.55	0.6	0
