# Supplementary material for: Coexistence of Lateral and Co-Tuned Inhibitory Configurations in Cortical Networks
Source: PLoS Comput Biol. 2011 Oct 6;7(10):e1002161. doi: 10.1371/journal.pcbi.1002161 (PMC3188483; doi:10.1371/journal.pcbi.1002161)
Supplement: Table S3 — Network parameters for the firing rate model. (PDF) [file pcbi.1002161.s009.pdf]

Table S3: Network parameters for the firing rate model:

	τ (ms)	ρ (cells/μm)	Synaptic transfer function (S)		
			M (Hz)	θ (nA)	n
Excitatory:	10	0.91	136	0.31	1.51
Inhibitory:	7.5	0.16	378	0.62	1.75
