# Supplementary material for: Coexistence of Lateral and Co-Tuned Inhibitory Configurations in Cortical Networks
Source: PLoS Comput Biol. 2011 Oct 6;7(10):e1002161. doi: 10.1371/journal.pcbi.1002161 (PMC3188483; doi:10.1371/journal.pcbi.1002161)
Supplement: Table S4 — Unitary response amplitudes for the firing rate model. (PDF) [file pcbi.1002161.s010.pdf]

Table S4: Unitary response amplitudes for the firing rate model:

	A (pA/Hz):
Thal → P:	0.52
Thal → FS:	0.81
P → P:	0.13
P → FS:	0.29
FS → P:	1.33
