# Supplementary material for: Coexistence of Lateral and Co-Tuned Inhibitory Configurations in Cortical Networks
Source: PLoS Comput Biol. 2011 Oct 6;7(10):e1002161. doi: 10.1371/journal.pcbi.1002161 (PMC3188483; doi:10.1371/journal.pcbi.1002161)
Supplement: Text S1 — Additional methods and results. (PDF) [file pcbi.1002161.s011.pdf]

Contents of supporting text:

A. Full model.

1. Model design

2. Robustness of spatiotemporal patterns of firing and synaptic conductances.
    a. Effects of noise
    b. Effects of background, spontaneous activity
    c. Effects of changing excitability of FS neurons

B. Calculation of Excitatory and Inhibitory Tuning in Feedforward model

1. Role of FS threshold in the transition between lateral inhibition and cotuning
2. Effects of differences in spatial inputs to excitatory and inhibitory cells

C. Simulations with non-Gaussian connectivity schemes.

References.

A. Full model

1. Model Design

Neuron model:
The adaptive exponential integrate and fire unit (aEIF; Naud et al., 2008) accurately reproduces the firing patterns of cortical neurons with relatively little computational cost, thereby allowing modeling large networks efficiently. The aEIF is governed by:

$$\frac{dV}{dt} = -\frac{1}{\tau_m}\left(\left(V - E_L\right) - \Delta_T \exp\left(\frac{V - V_T}{\Delta_T}\right) + g_L w - g_L I\right) , \qquad 1$$

$$\frac{dw}{dt} = \frac{1}{\tau_w}\left(a\left(V - E_L\right) - w\right) \qquad 2$$

The input conductances (g_L), membrane time constants (τ_m), voltage thresholds (V_T), afterhyperpolarization depths, and resting potentials were taken from experimental measurements (Table S1). The parameters governing the firing behaviors (a, b, τ_w, and Δ_T; Table S1) were adjusted so as to produce firing patterns and firing rate-current (F/I curves) that resembled those of the recorded P and FS cells.
Short-term depression and facilitation were implemented using a phenomenological model (Tsodyks and Markram, 1997; Tsodyks et al., 1998). The postsynaptic conductance amplitudes (A) evoked by the nth presynaptic spike when the previous spike occurred Δt ms earlier changes according to:

$$A_n = R_n u_n ,$$

$$R_{n+1} = R_n (1 - u_{n+1}) \exp(\frac{\Delta t}{\tau_{rec}}) + 1 - \exp(\frac{\Delta t}{\tau_{rec}}) ,$$

$$u_{n+1} = u_n \exp(\frac{\Delta t}{\tau_{fac}}) + U(1 - u_n \exp(\frac{\Delta t}{\tau_{fac}})) \qquad 3$$

The values for U, τ_{fac}, and τ_{rec} are given in Table S2.

2. Robustness of spatiotemporal patterns of firing and synaptic conductances.
Because of synaptic dynamics, which differ between the cell types, the net synaptic currents and the associated firing are likely to depend substantially both on the background activity of the network and on the temporal characteristics of the thalamic input. In the following, simulations were performed with the same network used in the main text but under different stimulus conditions. *The main finding is that while there were quantitative differences, the results are very similar to those presented in the main text. In particular:*

> The recurrent excitatory and inhibitory inputs to pyramidal cells were greatest at the onset of the stimulus.
> The thalamic and FS cell inhibitory input dominated the recurrent excitatory recurrent and non-FS inputs.
>Co-tuned configurations occurred with a spatially broad input and laterally inhibited configurations with narrow inputs.

<u>a. Effects of noise</u>

To examine the effects of background activity, sufficient white noise current (σ= 0.5, 0.75, 1 nA) was added to the neurons (Fig. S1) to produce voltage fluctuations (+/- 1 standard deviation) in P (FS) cells of 3.3 mV (4.0 mV), 5.2 (6.2), and 7.0 (7.5) respectively. P cells fired with a background rates of 0.1, 6, and 11 Hz for the 3 noise amplitudes. *The temporal profile of the conductances (Fig. S1B) remained qualitatively similar to those without noise (Fig. 2C-D of main text***.** *The spatial dependence of cotuning and lateral inhibition was maintained (Fig. S1C,D).*

<u>b. Effects of background, spontaneous activity</u>

Under *in vivo* conditions, the network may be spontaneously active. Background firing would cause tonic depression of the synaptic potentials, the degree of which differs between thal→P, thal→FS, P→P, P→FS, and FS→P connections. At steady state, the amplitudes of the various synaptic conductances would therefore differ from quiescent conditions. In Figure S2, the steady-state conductances were analytically calculated for all the synapses assuming that there was steady background firing of 10-40 Hz (Tsodyks et al. 1998). *The firing spatial profiles (Fig. S2A) were similar to those shown in figures 3 and 5 of the main text. The ratio of inhibitory to excitatory spatial widths did not change with the background activity (Fig. S2B).*

<u>c. Effects of changing excitability of FS neurons</u>

An important connection that was not included in the model was the inhibitory connections between FS neurons. The patterns of connections between these cell types are not yet known. To a first approximation, the major effects of these connections would be to reduce the responsiveness of FS cells to synaptic drive from the thalamus and other P cells. To simulate the reduced FS responsiveness, we raised the threshold of the FS cells from -47 mV to -37mV. Though the firing of FS cells decreased (compare Fig. S3C, left with S3B, left), the transition between cotuning and lateral inhibition was identical (S3B,C, middle, right). Similar results were obtained when the FS threshold was lowered to -52 mV (Fig. S3A).

B. Calculation of Excitatory and Inhibitory Tuning in Feedforward model

1. Role of FS threshold in transition from lateral inhibition to cotuning

The transition from lateral inhibition to cotuning, measured as a change in ratio of inhibitory to excitatory spatial widths W_{inh}/W_{exc} (Fig. 5C of main text), with increasing σ depends on the

presence of the FS firing threshold. This can be seen by substituting a linear transform for the threshold non-linearity in the input/output relation of the inhibitory neuron (Fig. S4A). Now, the shape of $F_{inh}(x)$ is exactly $I_{thal}(x)$. Because $F_{inh}(x)$ is convolved with $P_{inh}(x)$, $I_{inh}(x)$ is always broader than $I_{exc}(x)$. The difference is greatest when $I_{thal}(x)$ is very narrow (i.e. approaches a delta function) and least when $I_{thal}(x)$ is very broad (wider than $P_{inh}(x)$). However, the ratio asymptotically approaches 1 with increasing σ (Fig. S4B). Hence, the network always exhibits some degree of lateral inhibition. *The presence of a threshold process in the inhibitory cells lowers the σ value where the transition takes place.*

2. Effects of differences in spatial inputs to excitatory and inhibitory cells

For the calculation of excitatory and inhibitory tuning in the main text, the spatial spread of thalamic inputs to excitatory cells was assumed to be equal. For the following, the analysis in Fig. 5 of the main text (and equations 5a and 5b of the main text) are used to examine the N shift between cotuning and lateral inhibition when the spatial spread of thalamic inputs to excitatory ($P_{thal\text{-->} exc}(x)$) and inhibitory ($P_{thal\text{-->}inh}(x)$) neurons are not equal. The input from the thalamus is modeled as in equation 5b of the main text. I_{thal} is convolved with $P_{thal\text{-->} exc}(x)$ and $P_{thal\text{-->}inh}(x)$ to obtain the inputs to the excitatory ($I_{thal\text{->}exc}$) and inhibitory ($I_{thal\text{->}inh}$) cell populations.

$$P_{thal\rightarrow exc}(x) = (\frac{1}{sqrt(2\pi\sigma_{thal\rightarrow exc}{}^2)})\exp(-x^2/2\sigma_{thal\rightarrow exc}{}^2) \quad \text{4a}$$

$$P_{thal\rightarrow inh}(x) = (\frac{1}{sqrt(2\pi\sigma_{thal\rightarrow inh}{}^2)})\exp(-x^2/2\sigma_{thal\rightarrow inh}{}^2) \quad \text{4b}$$

In Figure S5, the width ratios are calculated for different values of$\sigma_{thal\text{-->}exc}$ and $\sigma_{thal\text{-->}inh}$ values. As the inhibitory input becomes broader (A to C), the region of perfect cotuning (w_{inh}/w_{exc} = 1) towards larger values of σ. However, the shift from lateral inhibition to cotuning still occurs.

C. Simulations with non-Gaussian connectivity schemes.

The simulations in the main text and above used a Gaussian connectivity profile which provided a good fit to experimental data (not shown), but the main findings are not sensitive to the choice of models for the connectivity profile, provided the inhibition is sufficiently strong. This is illustrated in figure S6, where three alternative models are used: uniform, i.e. boxcar-shaped (S6A), quadratic (inverted, rectified parabola, S6B), and asymmetric (derived from a binomial probability density function, S6C). In all three cases the shift between lateral inhibition and cotuning occurs (fig. S6A-C, left panels); likewise, there is a shift from sustained firing with narrow input (σ=40) to transient firing with narrow input (σ=160).

References:

Naud, R., Marcille, N., Clopath, C. & Gerstner, W. Firing patterns in the adaptive exponential integrate-and-fire model. *Biol Cybern* **99**, 335-347 (2008).

Tsodyks, M.V. & Markram, H. The neural code between neocortical pyramidal neurons depends

on neurotransmitter release probability. *Proc Natl Acad Sci U S A* **94**, 719-723 (1997).

Tsodyks, M., Pawelzik, K. & Markram, H. Neural networks with dynamic synapses. *Neural Comput* **10**, 821-835 (1998).
